# Angle-Dependent Dose Transformer Algorithm for Fast Proton Therapy Dose Calculations

**DOI:** 10.1016/j.ijpt.2026.101970

**Published:** 2026-10-08

**Authors:** Mikołaj Stryja, Danny Lathouwers, Zoltán Perkó

**Affiliations:** Faculty of Applied Science, Department of Radiation Science and Technology, Delft University of Technology, Mekelweg 5, Delft, 2628 CD, The Netherlands

**Keywords:** Deep-learning-based dose calculation, Pencil beam scanning proton therapy, Dose calculation, Deep learning

## Abstract

**Background:**

Accurate 3D dose calculation for Pencil Beam Scanning Proton Therapy (PBSPT) is typically performed using Monte Carlo (MC) dose engines. Although MC remains the gold standard for dosimetric accuracy, its high computational cost limits applicability in adaptive workflows and other tasks requiring repeated recalculation. Deep learning methods have recently emerged as a promising alternative for fast and accurate proton dose computation.

**Purpose and Methods:**

We address a major limitation of existing deep learning proton dose engines: the requirement that each beamlet be sampled in its beam’s-eye-view, with the proton beam direction orthogonal to the lateral image planes, which requires a local beam’s-eye-view resampling of the computed tomography (CT) patch for every beamlet. To overcome this, we propose the Angle-dependent Dose Transformer Algorithm (ADoTA), which augments the model with an additional input channel encoding a fast analytical beamlet shape projection. The projection supplies explicit beam direction information so that a single field-level CT transformation remains: the CT is rotated and resampled once per irradiation field to an isotropic 2 × 2 × 2 mm^3^ grid, and each beamlet is then obtained by cropping the relevant sub-volume from this shared grid. The model was trained on CT data from 108 patients to predict beamlet dose distributions for initial energies from 70 to 150 MeV (thoracic) and 70 to 270 MeV (abdominal/pelvic) over an 80 × 110 mm^2^ field and evaluated on an independent, non-overlapping test cohort of 50 patients.

**Results:**

On the independent test set, ADoTA achieved gamma pass rates of 99.40% ± 0.86% and 99.87% ± 0.23% for thoracic and abdominal/pelvic cases, respectively, under the Γ(1%, 3 mm, 0.1%) criterion. Batched inference achieved an effective throughput equivalent to 3.14 ± 0.31 ms per beamlet at a batch size of 200, whereas a beamlet processed on its own required 14.1 ms. By resampling each field once to a 2 × 2 × 2 mm^3^ grid and then only cropping the relevant sub-volume per beamlet, ADoTA avoids the beamlet-specific local beam’s-eye-view resampling required by prior methods. In a 5640-spot prostate-plan benchmark with both routes optimized on the graphics processing unit (GPU), the analytical beamlet shape projection was 3–4× cheaper on compute and approximately 6–7× cheaper including host-device transfer than batched local beam’s-eye-view sampling, per beamlet, reducing the plan’s direction-encoding cost by 9.6× and its total compute time by 1.48×. For full treatment plans Γ(2%, 2 mm, 10%) pass rates averaged 95.1% for lung plans and 96.1% for prostate plans, while the target near-minimum dose *D*_98%_ was systematically underestimated by 1.8% to 2.5% of prescription in prostate plans and by 4.2% to 5.9% in thoracic plans.

**Conclusions:**

ADoTA is a beamlet angle-aware deep learning proton dose engine that avoids beamlet-specific local beam’s-eye-view resampling, retaining a single field-level CT transformation. It achieves close to Monte Carlo-level accuracy at the beamlet level and high accuracy at the full-plan level, with a clinically relevant target underdosage in thoracic plans.

## Introduction

Accurate 3-dimensional dose calculation is central to radiotherapy (RT) safety and efficacy and is particularly demanding in pencil beam scanning proton therapy, where steep gradients and range sensitivity require precise modeling of proton energy deposition.^1^ In clinical workflows, CT-based patient geometry and objectives define a set of candidate proton spots (positions and energy layers), the dose-influence matrix is computed, and inverse planning optimizes the non-negative spot weights such that the final dose is the weighted superposition of spot-wise beamlets.

The accuracy of the calculated 3-dimensional dose distribution in pencil beam scanning proton therapy is particularly critical because proton dose is highly sensitive to anatomical heterogeneities as well as range and setup errors.^2^, ^3^, ^4^, ^5^ Monte Carlo (MC) simulation remains the reference standard because it explicitly models stochastic proton interactions in patient-specific media,^6^ but this accuracy is computationally expensive,^7^ with beamlet-level runtimes that can be prohibitive for large-scale recalculation (eg, 43.6 seconds per beamlet for 10^7^ particles^8^). Faster yet accurate beamlet dose calculation is a persistent need in (i) online/adaptive replanning^9^ and (ii) robust planning and evaluation across many uncertainty scenarios,^10^, ^11^ where the dose-influence matrix *d*_*ij*_ must be constructed and repeatedly recomputed for thousands of beamlets under multiple scenarios, making dose calculation a dominant bottleneck.

Clinically, the pencil beam algorithm (PBA) is widely used as a fast dose engine for planning and optimization,^12^ but its accuracy degrades in heterogeneous anatomy (eg, thorax and head-and-neck).^13^ Multiple extensions have been proposed to mitigate these limitations.^14^, ^15^, ^16^, ^17^ Notably,^18^ reports GPU PBA implementations targeting the Compute Unified Device Architecture (CUDA) framework.^19^ In parallel, GPU-accelerated MC-like dose engines have been developed.^20^, ^21^, ^22^, ^23^, ^24^ Although these advances substantially reduce computation time, sub-second dose calculation time methods for repeated beamlet recalculation remain desirable for adaptive and robust workflows, motivating deep learning approaches for near-real-time dose computation.

Early deep learning studies in proton therapy typically addressed surrogate tasks, such as denoising MC doses^25^ or predicting clinically acceptable plan doses from CT and structures.^26^ More recent work targets beamlet-level dose engines that aim to learn proton transport from patient geometry. Neishabouri et al^27^ formulated monoenergetic beamlet dose computation as sequence modeling along the beam path using a long short-term memory (LSTM) network.^28^ Pastor-Serrano and Perkó ^8^ proposed the Dose Transformer Algorithm (DoTA), a transformer-based general beamlet dose computation model that conditions on beam energy. Pang et al^29^ introduced the Multi-Energy Dose LSTM (MED-LSTM) to enable multi-energy inference within an LSTM framework. Neishabouri et al^30^ later proposed CC-LSTM, combining convolutional neural network feature extraction with a convolutional LSTM (ConvLSTM) pathway to better preserve spatial coherence along depth.

Existing beamlet-level deep learning dose engines require each beamlet to be centered in the input patch and perpendicular to the sampling grid.^8^, ^27^, ^29^, ^30^ For off-axis spots, this requirement implies a local beam’s-eye-view resampling of the CT patch for every beamlet before prediction, followed by remapping of the predicted dose for accumulation. Our contribution removes that beamlet-specific step: instead of resampling a CT patch per spot, we encode beamlet orientation through an analytical projection channel defined on a shared field-aligned grid so that the only geometric transformation retained is a single field-level CT resampling.

## Methods and materials

### Ground truth data generation

#### Data sources

A total of 108 CT scans were collected from two publicly available datasets of the Cancer Imaging Archive (TCIA): 92 thoracic scans from LUNG-PET-CT-Dx^31^ and 16 abdominal/pelvic scans from the Stage II Colorectal CT collection.^32^ Only CT volumes with in-plane resolution < 1.5 mm and slice thickness ≤2 mm were included to support resampling to an isotropic 1 mm^3^ grid. For model development, we performed a patient-level stratified split by anatomy, allocating 80% of thoracic and 80% of abdominal/pelvic scans to training and the remaining 20% of each group to validation.

Two independent test cohorts were defined. First, the beamlet-level test set comprised 20 additional thoracic scans (LUNG-PET-CT-Dx) and 30 additional abdominal/pelvic scans (Stage II Colorectal), all excluded from training and validation. The 3500 beamlets evaluated in Section “Results” are drawn from these 50 scans, approximately 70 per patient, so they are clustered within patients and are not independent observations. Beamlets from the same patient share that patient’s anatomy, but how similar they are is unknown: every record draws its own isocenter, gantry angle, spot position, deflection angles, and energy, so two beamlets from one patient can cross completely different tissue. The generation pipeline did not record which patient each beamlet came from, so patient-clustered statistics cannot be computed retrospectively, and the per-beamlet statistics reported throughout describe the evaluated beamlet population rather than 50 independent samples. Second, a full-plan test set with expert delineations was used for plan-level dose validation, using prostate cases from the Prostate Anatomical Edge Cases collection^33^ and lung cases from NSCLC Radiomics.^34^ Treatment plans were generated in OpenTPS^35^ using a standardized intensity-modulated proton therapy procedure. Plans targeted 60 Gy(RBE), where RBE denotes relative biological effectiveness, with target margins of 3.5 mm for prostate and 1.5 mm for thoracic plans, planning target volume coverage constrained to 58 to 62 Gy, and maximum-dose constraints of 5.0 Gy applied to Bladder and Rectum (prostate) and Spinal Cord (lung).

#### Training sample definition

To train and validate the model, we constructed a dataset $D={\left\{r_{i}\right\}}_{i=1}^{N}$ with *N* = 70 285 records. Each record *r*_*i*_ = {**V**_*i*_, **Φ**_*i*_, *ϵ*_*i*_, **D**_*i*_} consisted of a 3D CT volume $V_{i}\in R^{D\times H\times W}$, a fast projection of the beamlet trajectory $\Phi_{i}\in R^{D\times H\times W}$, the mean proton beamlet energy $\epsilon_{i}\in R_{+}$, and the corresponding Monte Carlo dose $D_{i}\in R^{D\times H\times W}$ computed with MCsquare,^36^ where *D*, *H*, and *W* denote the depth, height, and width of the parent volume of interest (VOI), and *d*, *h*, and *w* denote the corresponding dimensions of the cropped sub-volumes used by the model.

#### Beamlet sampling and Monte Carlo

Each record was generated using in-house software. The CT was loaded from its DICOM series and resampled to an isotropic 1 mm^3^ grid. For every simulation, an isocenter position ISO was randomly sampled within the patient body region, and the grid was rotated about the patient *z*-axis through ISO by a gantry angle $\alpha_{G}\sim U\left(0^{\circ },360^{\circ }\right)$, aligning it to the field direction. This gantry rotation provides the field-level alignment and is the only grid reorientation in the pipeline, since the model conditions on the beamlet direction through the analytical projection rather than through a per-beamlet CT rotation. A virtual source position *S*_v_ was placed upstream of ISO at the nozzle-to-isocenter distance *D*_nozzle_ specified in the Beam Data Library (BDL). The beamlet trajectory was defined by sampling a spot position (SP_*x*_, SP_*y*_) in the isocenter plane relative to ISO, with deflection angles constrained to −2.5^∘^ < *θ*_*x*,*y*_ < 2.5^∘^ to keep the ray within the CT image bounds and intersecting the patient body contour. For each beamlet, 10^7^ particles were simulated with MCsquare on this field-aligned 1 mm^3^ grid, yielding a statistical uncertainty < 0.5%. For each spot position, two energies were independently sampled from a uniform distribution $U\left(\epsilon_{\min},\epsilon_{\max}\right)$, with $\epsilon_{\min}=70\text{MeV},\epsilon_{\max}=150\text{MeV}$ for thoracic cases, and $\epsilon_{\max}=270\text{MeV}$ for abdominal/pelvic cases. The same per-anatomy energy sampling distribution governs both the training and the validation cohorts, which differ only in patient identity per the 80/20 patient-level split defined under *Data sources* (Section “Ground Truth Data Generation”), and is reused for the independent beamlet-level test set (Table 1). Plan-test energy ranges, by contrast, are determined by OpenTPS plan optimization and are reported per plan in Table 2a.

**Table 1:** Beamlet-level comparison of ADoTA with published proton dose calculation models.

Metric	ADoTA (this work)	CC-LSTM Neishabouri et al^30^	DoTA Pastor-Serrano and Perkó^8^	MED-LSTM Pang et al^29^
Cohort and energy	(1) Thoracic, [70, 150] MeV (1400 beamlets) (2) Abd./pelvic, [70, 270] MeV (2100 beamlets)	(1) Thoracic, 48.1 MeV (2) Thoracic, 79.17 MeV (3) Thoracic, 102.6 MeV	(1) Thoracic, [70, 140] MeV (2) Head & neck, [70, 140] MeV (3) Prostate, [70, 220] MeV	(1) Thoracic, [69, 114.5] MeV (2) Nasopharynx, [69, 161.4] MeV (3) Prostate, [114.5, 200.8] MeV
Γ(1%, 3 mm, 0.1%) [%]	(1) 99.40±0.86 (2) 99.87±0.23	–	(1) 99.46±0.81 (2) 99.21±1.23 (3) 99.51±1.46	–
Γ(1%, 3 mm, 10%) [%]	(1) 98.47±1.33 (2) 99.46±0.63	–	–	(1) 99.93±0.06 (2) 99.81±0.32 (3) 99.89±0.12
Γ(2%, 2 mm, 10%) [%]	(1) 98.33±1.83 (2) 99.63±0.60	–	–	(1) 99.97±0.03 (2) 99.92±0.19 (3) 99.96±0.07
Γ(1%, 2 mm, 3%) [%]	(1) 97.86±2.30 (2) 99.46±0.72	(1) 94.28±1.76 (2) 97.21±0.84 (3) 97.83±0.91	–	–
MAPE [%]	(1) 5.71±1.91 (2) 4.46±1.05	–	–	–
Δ*R*_100_ [mm]	(1) −0.94±2.32 (2) −0.49±0.79	–	–	–
Δ*R*_80_ [mm]	(1) 0.39±5.35 (2) −0.28±0.76	–	–	–
Δ*R*_20_ [mm]	(1) −0.12±3.49 (2) −0.22±0.58	–	–	–
ΔDFW [mm]	(1) −0.51±5.80 (2) 0.06±0.79	–	–	–

For each model, the first data row lists the test cohort(s) and energy range(s) reported in the corresponding study; numbered metric entries refer to those cohort/energy entries. ADoTA results are reported on our 3500-beamlet test set (1400 thoracic and 2100 abdominal/pelvic beamlets; Section “Ground Truth Data Generation”). Γ pass rates, MAPE, and range metrics (Δ*R*_100_, Δ*R*_80_, Δ*R*_20_, and ΔDFW) are reported as mean ± standard deviation.

**Table 2:** (a) Plan-level runtime and dose agreement of ADoTA relative to the MCsquare dose engine. (b) Benchmark comparison of ADoTA with published deep-learning-based proton dose calculation methods and a GPU-based pencil beam algorithm.

**(a)**								
Plan Identifier	Prostate				Thoracic			
	1	2	3	4	1	2	3	4
Spots	4820	9273	1403	2897	285	40	2055	5019
Fields	2	2	2	2	3	1	3	3
Target prescription [Gy(RBE)]	60	60	60	60	60	60	60	60
Energy range [MeV]	[147, 197]	[160, 193]	[128, 190]	[130, 192]	[124, 155]	[119, 137]	[81, 157]	[109, 176]
*ADoTA pipeline stage [s]*								
Per-field rotation	0.16	0.14	0.14	0.21	0.30	0.05	0.35	0.37
Cropping + shape projection	11.38	20.99	3.41	7.17	0.87	0.28	5.07	13.50
Input preparation	3.98	7.50	1.06	2.22	0.26	0.05	1.49	4.01
Network inference	12.55	15.50	2.79	5.23	0.95	0.62	3.83	8.68
Postprocessing + deposition	3.05	6.04	0.99	2.04	0.18	0.03	1.53	3.99
Per-field de-rotation	1.08	0.99	0.87	1.17	1.34	0.48	1.88	2.10
Dose write-out	0.55	0.44	0.36	0.60	0.38	0.38	0.24	0.23
*Total ADoTA [s]*	33.22	52.11	9.98	19.15	4.64	2.19	14.93	33.46
*Per beamlet [ms]*	6.89	5.62	7.11	6.61	16.27	54.66	7.26	6.67
MC *d*_*ij*_ [s]	1400.71	2919.93	578.70	1674.88	72.26	23.49	787.45	1371.40
MC full plan [s]	1258.54	1216.77	2181.39	1727.93	1342.71	491.60	1959.16	1411.08
Γ(2%, 2 mm, 10%) [%]	94.8	96.8	96.3	96.5	97.7	98.1	91.2	93.4
Γ(3%, 3 mm, 10%) [%]	98.5	99.4	99.1	99.3	99.6	99.3	97.7	97.2
Δ*D*_98%_ (target) [%]	−1.8	−2.5	−1.9	−2.0	−5.9	−5.4	−5.2	−4.2
mean ∣Δ*D*_2%_∣ (OAR) [%]	5.0	3.8	2.0	3.0	0.1	0.8	1.4	0.5

**(b)**						
Model	Site	Energy (MeV)	Mean (%)	Std (%)	Min (%)	Max (%)
DoTA^c^ Pastor-Serrano and Perkó^8^	Thoracic	[70, 220]	99.69	0.04	99.64	99.73
	Head & neck	[70, 220]	99.63	0.20	99.39	99.88
	Prostate	[70, 220]	99.77	0.05	99.71	99.82
MED-LSTM Pang et al^29^	Thoracic	Unspecified	99.42	0.17	–	–
	Nasopharynx	Unspecified	98.38	0.12	–	–
	Prostate	Unspecified	99.97	0.02	–	–
ADoTA (this work)	Thoracic	[81, 176]^b^	95.10	2.91	91.20	98.10
	Prostate	[128, 197]	96.10	0.77	94.80	96.80
DoseCUDA Bhattacharya et al,^18^	Avg (4 sites)^a^	[70.2, 228.7]	96.0	5.1	–	–

(a) Plan compute time (excludes disk I/O and data loading) of the ADoTA full-plan reconstruction pipeline, with analytical beamlet shape projection and network inference evaluated on GPU, broken down by stage for 4 prostate and 4 thoracic treatment plans. Model load (0.27 s) and plan load (0.55 s) are constant across plans and are excluded from the per-plan totals. The Monte Carlo *d*_*ij*_ (10^4^ primaries per beamlet) and full-plan (10^8^ primaries per field) reference times are reported for comparison. Accuracy is quantified by Γ(2%, 2 mm, 10%) and Γ(3%, 3 mm, 10%) pass rates relative to the full-plan MC reference. Δ*D*_98%_ and ∣Δ*D*_2%_∣ are extracted from the dose-volume histograms of the ADoTA and MC doses; a detailed per-plan breakdown, including absolute values, *D*_95%_, *D*_2%_, and signed per-organ results by anatomical name, is given in Appendix A.10. (b) Comparative performance summary of ADoTA (this work) against published dose calculation methods (DoTA,^8^ MED-LSTM,^29^ and DoseCUDA^18^). Results are stratified by anatomical site and proton beam energy range. For each model, the table reports the descriptive statistics (Mean, Standard Deviation, Minimum, and Maximum) of the gamma pass rates achieved on the respective test sets. The evaluation metric uses a passing criterion of Γ(2%, 2 mm) with a 10% low-dose threshold.

a Aggregate mean across prostate, esophagus, brain, and thoracic cases.

b Thoracic plan energies (up to 176 MeV) exceed the beamlet-level thoracic training range of [70, 150] MeV; see Section “Methods and Materials” and Appendix A.12.

c DoTA plan results were reported for plans assuming no beamlet scanning, with normally incident beamlets and wide targets covered by isocenter shifts.

To introduce additional angular variability for the same target spot and underlying heterogeneity, the virtual source was translated by $\overset{\to }{T}=\left[t_{x},t_{y}\right]$, where $t_{x},t_{y}\sim U\left(-40,40\right)$ mm, yielding a secondary source position $S\prime_{v}$ and incidence angles $\theta \prime_{x}$ and $\theta \prime_{y}$ while keeping the CT geometry unchanged (Figure 1a-b). For each trajectory (nominal and translated), the beamlet shape projection **Φ** was computed by transforming each voxel center **u** = (*x*, *y*, *z*) into a beamlet-aligned frame u′=(x′,y′,z′) via rotation matrices **R**_*y*_(*θ*_*x*_) and **R**_*x*_(*θ*_*y*_):

(1) $$\left[\begin{array}{c} x\prime \\ y\prime \\ z\prime \end{array}\right]=R_{y}\left(\theta_{x}\right)R_{x}\left(\theta_{y}\right)\left(\left[\begin{array}{c} x\\ y\\ z \end{array}\right]-u_{0}\right),$$

where **u**_0_ denotes the beamlet entrance point into the CT grid. The same formulation applies for the translated source by substituting (*θ*_*x*_, *θ*_*y*_) with $\left(\theta \prime_{x},\theta \prime_{y}\right)$. The projection was modeled as a 2D Gaussian in the lateral coordinates:

(2) $$\Phi \left(u\right)=\frac{1}{2\pi \sigma_{x}\left(\epsilon \right)\sigma_{y}\left(\epsilon \right)}\exp\left(-\frac{{\left(x\prime \right)}^{2}}{2\sigma_{x}{\left(\epsilon \right)}^{2}}-\frac{{\left(y\prime \right)}^{2}}{2\sigma_{y}{\left(\epsilon \right)}^{2}}\right),$$

where *σ*_*x*_(*ϵ*) and *σ*_*y*_(*ϵ*) are the energy-dependent in-air spot sizes at the nozzle exit, taken from the same BDL used by MCsquare. Although the in-air spot is measured near the isocenter during commissioning, the BDL already stores these spot sizes extrapolated to the nozzle exit. The BDL follows a single-Gaussian beam model. The BDL also specifies, for each energy, the beam divergence and its correlation with the spot size, which define the in-air phase space used by MCsquare for ground-truth simulation but are not encoded in **Φ**. Thus, **Φ** provides only the beamlet axis and energy-dependent in-air spot size. Patient-specific transport effects such as scattering, range perturbations, and heterogeneity effects are learned from the Monte Carlo dose labels. For each trajectory and energy, MCsquare dose was computed on the full resampled CT grid and then cropped to a fixed-size volume of interest (VOI) centered around the Bragg peak region (a 400 × 80 × 80 voxel volume at 1 mm^3^).

**Figure 1:**
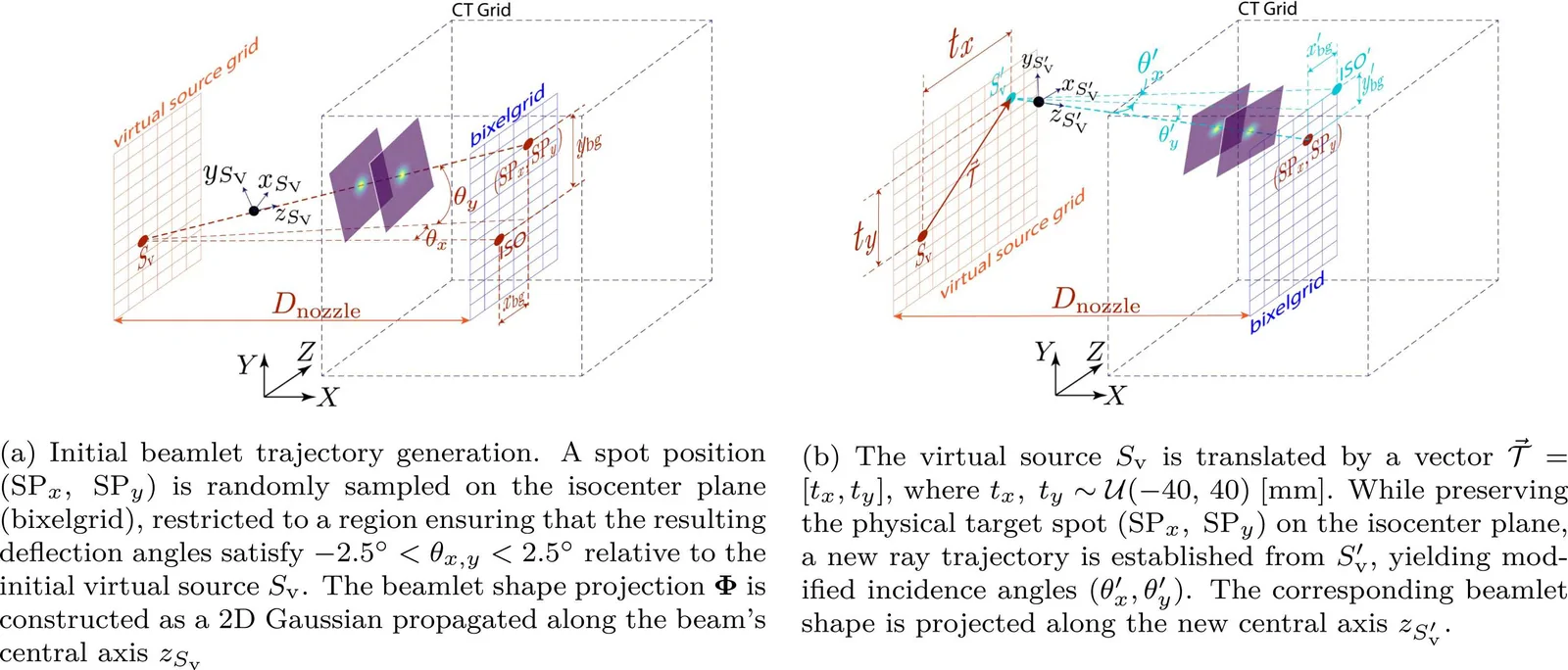
Sampling process for training records *r*_*i*_. For each spot position (SP_*x*_, SP_*y*_), two virtual source positions are defined: the nominal *S*_v_ and the translated $S\prime_{v}$. For each source position, simulations are performed at two distinct proton energies. This configuration ensures that the dataset encompasses both angular and energy variability.

### Data augmentation technique

The fixed-size VOI used during data generation was intentionally selected larger than required for most beamlets, providing spatial margin for translational augmentation. Based on the defined energy range and angular constraints, we empirically found that a reduced extent of 320 × 60 × 60 mm^3^ captures the dose deposition for the vast majority of beamlets. Accordingly, during training, and inference the model operates on random sub-crops $V_{i,\mathrm{crop}},\Phi_{i,\mathrm{crop}},D_{i,\mathrm{crop}}\in R^{d\times h\times w}$ of size 320 × 60 × 60 mm^3^ extracted from the parent VOI, which are then resampled to 2 × 2 × 2 mm^3^ using trilinear interpolation.

#### Translational cropping

The crop center was shifted in the beam’s-eye-view (BEV) plane by (Δx′,Δy′) in beamlet-aligned lateral coordinates, with Δx′,Δy′~U(−5,5) mm. A shifted crop was accepted only if the fractional dose FD retained inside the crop was at least 99% of the total deposited dose:

(3) $$\mathrm{FD}=\frac{\sum_{k}^{n_{v,\mathrm{crop}}}D_{i,{\mathrm{crop}}_{k}}}{\sum_{k}^{n_{v}}D_{i_{k}}}\geq 0.99,$$

where *n*_*v*,crop_ and *n*_*v*_ represent the number of voxels of the cropped sub-volume **D**_*i*,crop_ and the original dose distribution **D**_*i*_. If the criterion was not met after a fixed maximum number of attempts, the unshifted crop was used.

#### In-plane rotations

A discrete in-plane rotation *ϕ* ∈ {0^∘^, 90^∘^, 180^∘^, 270^∘^} was applied around the depth axis consistently to **V**_*i*,crop_, **Φ**_*i*,crop_, and **D**_*i*,crop_.

### Model Architecture: Angle-dependent Dose Transformer Algorithm (ADoTA)

The proposed model, ADoTA, adapts the hybrid architecture of Pastor-Serrano et al^37^ to proton beamlet dose prediction. It follows a 3D U-Net backbone^38^ with a transformer encoder bottleneck and residual/skip connections.^39^ In contrast to the photon setting, where a secondary input encodes the multileaf collimator shape, ADoTA uses this pathway to encode the beamlet trajectory via a Gaussian projection **Φ** parameterized by in-air spot sizes at the nozzle exit (Figure 2).

**Figure 2:**
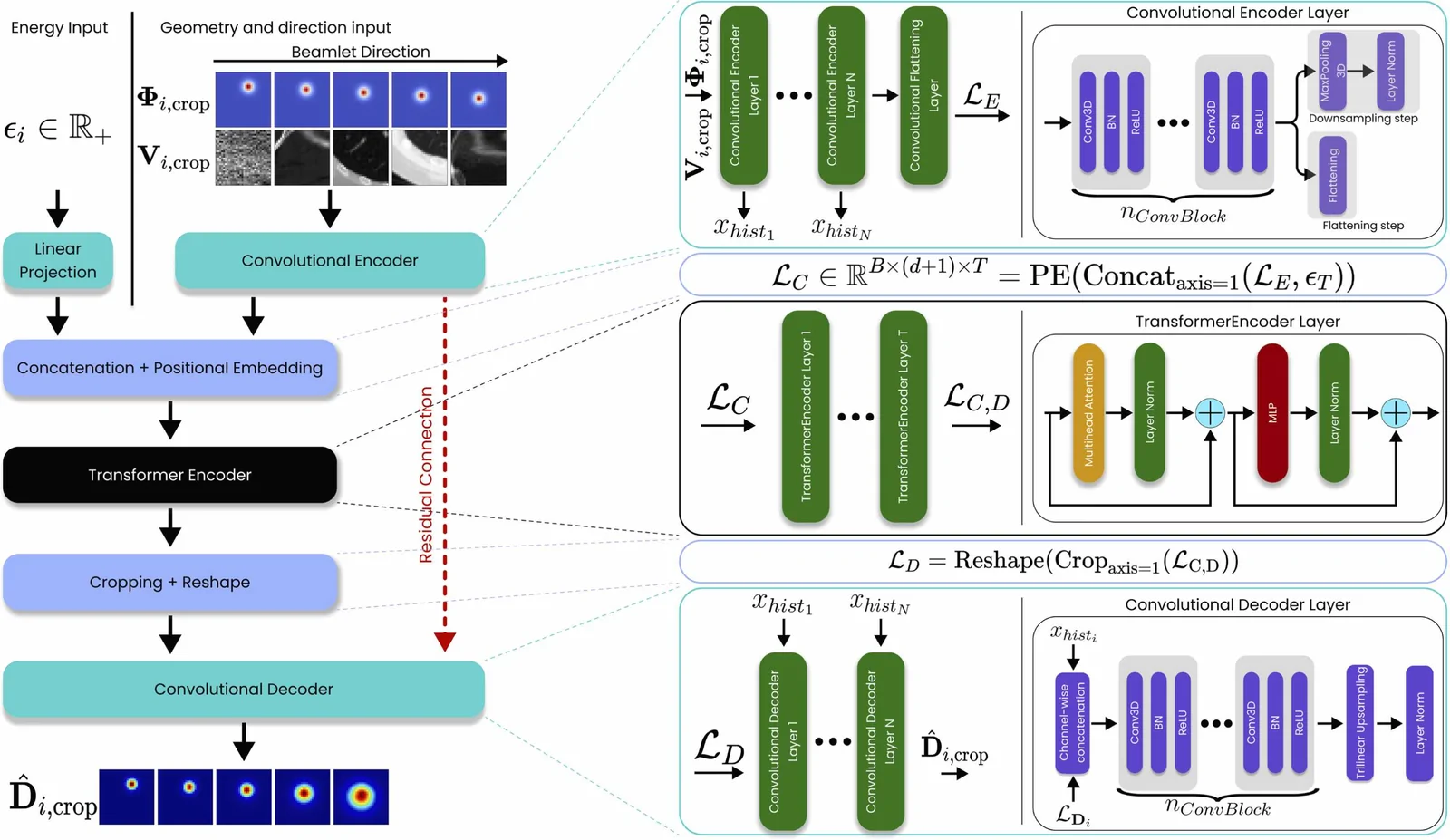
Overview of the Angle-dependent Dose Transformer Algorithm (ADoTA). The model takes as input a concatenated batch of CT grids and beamlet shape projection representations (**V**_crop_∣**Φ**_crop_), together with the corresponding batch of proton beamlet mean energies, ***ϵ***. The encoder generates the latent space $L_{E}$, which is conditioned by energy token *ϵ*_*T*_ to form the energy-conditioned latent space $L_{C}$. After positional embedding and transformer encoding, the output $L_{\mathrm{C,D}}$ is reshaped and cropped into the 3D latent volume $L_{D_{\mathrm{cropp}}}$. The decoder, connected to the encoder via residual connections,^39^ reconstructs the spatially aligned batch of dose distributions ${\overset{ˆ}{D}}_{\mathrm{crop}}$. Figure represents the processing of the batch element.

ADoTA takes as input a tensor $x\in R^{B\times 2\times d\times h\times w}={\mathrm{Concat}}_{\mathrm{ch}}\left(V_{\mathrm{crop}},\Phi_{\mathrm{crop}}\right)$, together with a secondary energy input $\epsilon \in R^{B\times 1}$. The network outputs ${\overset{ˆ}{D}}_{\mathrm{crop}}\in R^{B\times 1\times d\times h\times w}$.

#### Convolutional encoder and tokenization

The encoder consists of *N* stages, each formed by *n*_ConvBlock_ convolutional blocks (Conv3D + batch normalization^40^ + rectified linear unit (ReLU)^41^). After each stage, anisotropic max pooling (1 × 2 × 2) is applied, followed by layer normalization.^42^ All encoder convolutions use 64 feature channels with 3 × 3 × 3 kernels. In the convolutional flattening module (Figure 2), two additional Conv3D layers reduce the channel dimension from 64 to $e_{\mathrm{features}}$. A slice-wise flattening operation then collapses the in-plane spatial dimensions while preserving the depth axis, yielding a latent sequence $L_{E}\in R^{B\times d\times T}$, where *T* is the token dimension.

#### Energy conditioning and transformer bottleneck

Beam energy conditioning is implemented by projecting the scalar *ϵ* to a *T*-dimensional embedding via a linear layer, producing an energy token $\epsilon_{T}\in R^{B\times 1\times T}$. This token is prepended to the depth-wise token sequence: $L_{C}={\mathrm{Concat}}_{\mathrm{depth}}\left(\epsilon_{T},L_{E}\right)\in R^{B\times \left(d+1\right)\times T}$. Interpretable in proton therapy terms, this token provides global conditioning on the initial beam energy, that is, the primary determinant of range and the location/shape of the distal fall-off. A learned positional embedding PE is added elementwise along the depth dimension.

The transformer bottleneck comprises *n*_trans_ stacked transformer encoder layers.^43^ Each layer includes multi-head self-attention followed by a two-layer feed-forward multilayer perceptron (MLP) with ReLU activation, with residual connections and layer normalization applied after each sub-module. The output is $L_{C,D}\in R^{B\times \left(d+1\right)\times T}$.

#### Decoder

After transformer processing, the prepended energy token is removed to restore a length-*d* sequence, which is reshaped back to a 3D latent volume $L_{D}$. The decoder mirrors the encoder: each decoding stage upsamples the features, concatenates them with the corresponding encoder features via skip connections, and applies *n*_ConvBlock_ convolutional blocks. Two final 3D convolutions map features to a single output channel, producing ${\overset{ˆ}{D}}_{\mathrm{crop}}$.

#### Hyperparameter selection

We performed a grid search over *n*_ConvBlock_ ∈ {1, 2}, $e_{\mathrm{features}}\in \left\{4,16,32,64\right\}$, *n*_trans_ ∈ {1, 2}, and *N* ∈ {3, 4}. The selected configuration was *N* = 4, *n*_ConvBlock_ = 1, *n*_trans_ = 1, and $e_{\mathrm{features}}=32$, with 4 attention heads and MLP hidden size 128.

### Loss function

Proton dose prediction is highly sensitive to range errors. To penalize discrepancies in the longitudinal depth–dose behavior, we augment a voxel-wise mean squared error (MSE) term with an Integral Depth Dose (IDD) loss:^5^

(4) $$L_{\mathrm{total}}=\lambda \;L_{\mathrm{MSE}}+\left(1-\lambda \right)\;L_{\mathrm{IDD}},\;\lambda \in \left[0,1\right],$$

where *λ* = 0.7 was selected via grid search.

Let ${\overset{ˆ}{D}}_{\mathrm{crop}},D_{\mathrm{crop}}\in R^{d\times h\times w}$ denote the predicted and MC 3D dose distributions, respectively. We define

(5) $$L_{\mathrm{MSE}}\left({\overset{ˆ}{D}}_{\mathrm{crop}},D_{\mathrm{crop}}\right)=\frac{∥{\overset{ˆ}{D}}_{\mathrm{crop}}-D_{\mathrm{crop}}{∥}_{2}^{2}}{∥D_{\mathrm{crop}}{∥}_{2}^{2}},$$

and the IDD profile at depth index *k* as

(6) $$\mathrm{IDD}\left(D,k\right)=\sum_{i=1}^{h}\sum_{j=1}^{w}D_{k,i,j}\;\Delta x\;\Delta y,$$

with pixel spacing Δ*x* and Δ*y*. The IDD loss is

(7) $$L_{\mathrm{IDD}}=\frac{\sum_{k=1}^{d}{\left(\mathrm{IDD}\left({\overset{ˆ}{D}}_{\mathrm{crop}},k\right)-\mathrm{IDD}\left(D_{\mathrm{crop}},k\right)\right)}^{2}}{\sum_{k=1}^{d}\mathrm{IDD}{\left(D_{\mathrm{crop}},k\right)}^{2}}.$$

Both $L_{\mathrm{MSE}}$ (Equation (5)) and $L_{\mathrm{IDD}}$ (Equation (7)) are scalar values computed per sample and combined as in Equation (4) with *λ* = 0.7. During training, $L_{\mathrm{total}}$ is reduced by taking its mean over the mini-batch.

### Training details

ADoTA was trained using the composite loss function defined in Equation (4), optimized with the AdamW optimizer^44^ and a weight decay of 10^−3^. Training is performed with a batch size of *B* = 56. The initial learning rate was set to 10^−3^. We utilized the ReduceLROnPlateau scheduler to decay the learning rate by a factor of 0.75 whenever the validation loss stagnated for more than 20 epochs (patience).

To enhance generalization, we apply data augmentation techniques described in detail in Section “Data Augmentation Technique.” Training was set to run for up to 400 epochs but utilized early stopping with a patience of 100 epochs to prevent overfitting. All experiments were carried out on an NVIDIA A40 GPU using the PyTorch framework.^45^

We performed an ablation study with 5 configurations. The reference one was the full ADoTA model trained with the MSE + IDD objective (*λ* = 0.7). To isolate the contribution of the IDD term, we trained an otherwise identical model with *λ* = 1 in Equation (4), corresponding to pure voxel-wise MSE. To isolate the contribution of the encoder–decoder convolutional residual connections, we trained a third model with the full MSE + IDD objective but with these residual connections removed. To test the specific value of the analytical projection **Φ** (Equation (2)) against simpler direction encodings, we trained a fourth and fifth model in which **Φ** was replaced by two encodings sharing the same beamlet centerline: a fixed-width Gaussian tube of the dataset-median spot size (**Φ**^*E*^), and a binary indicator of the centerline itself, marking at each depth the single lateral voxel nearest the central ray (**Φ**^*F*^); both are defined in Appendix A.3. Apart from the intended ablated component, the training data, data augmentation, optimizer, scheduler, early-stopping criterion, and checkpoint selection were unchanged across all 5 configurations. For all configurations the reported checkpoint was selected by the lowest validation mean absolute percentage error (MAPE), and all 5 configurations are reported with MAPE and the IDD-derived range metrics Δ*R*_80_, Δ*R*_20_, and the distal fall-off width error ΔDFW, all defined in Section “Model evaluation.” The independent test set was not used for this comparison.

### Model evaluation

We benchmarked the performance of our model on the test set against published data from the DoTA,^8^ the spatio-temporal mono-energetic dose calculation model (CC-LSTM),^30^ the multi-energy dose LSTM (MED-LSTM)^29^ and the PBA.^18^ To quantify model performance, we used the gamma-index metric Γ, introduced by Low et al.^46^ Since these methods reported their results using different test sets and gamma criteria, in this work we performed evaluations using a set of distinct Γ criteria for consistency.

For comparison with DoTA,^8^ we adopted a 1% dose threshold and a 3 mm distance-to-agreement (DTA), with a dose cutoff at 0.1%. For comparison with the CC-LSTM^30^ we used a 1% dose threshold, a 2 mm distance-to-agreement, and a 3% dose cutoff. Finally, to compare against the MED-LSTM,^29^ we used Γ(1%, 1 mm) and Γ(2%, 2 mm) with a dose cutoff at 10%. In the full treatment plan comparison, we applied Γ(2%, 2 mm) with a 10% dose cutoff.

#### Gamma pass rate

All gamma analyses were performed using the PYMEDPHYS library, version 0.40.0.^47^ A global gamma criterion was used for every gamma computation reported in this work, including the selection of representative cases in Section “Results,” with the dose-difference threshold normalized to the maximum reference dose. For the beamlet-level analysis, gamma was computed on the model’s native 2 mm isotropic grid, with the reference and evaluated distributions defined on identical spatial axes and no resampling applied. To allow sub-voxel distance-to-agreement evaluation on this 2 mm grid, PYMEDPHYS internally interpolates both distributions onto a grid ten times finer than the voxel spacing, giving a search resolution of 0.2 mm. For the plan-level analysis, where the full-plan MCsquare reference dose is natively computed on a 1 mm isotropic grid, the predicted dose was resampled from its native 2 mm grid to this 1 mm grid by trilinear interpolation, and gamma was computed on the 1 mm grid. The specific dose threshold (0.1%, 3%, or 10%) is denoted explicitly in each gamma criterion as Γ(dose%, DTA mm, cutoff%) throughout this work.

#### Auxiliary global metrics

As a supportive metric, the average relative error *ρ*, the root mean square error (RMSE), and MAPE were used to explicitly compare dose differences between dose distributions. For the dose predicted by our model ${\overset{ˆ}{D}}_{\mathrm{crop}}$ and the reference dose distribution **D**_crop_, with *n*_*v*_ = *d* × *h* × *w* voxels, *ρ*[%] is defined as

(8) $$\rho =\frac{1}{n_{v}}\frac{||D_{\mathrm{crop}}-{\overset{ˆ}{D}}_{\mathrm{crop}}|{|}_{1}}{\max\left(D_{\mathrm{crop}}\right)}\times 100\left[\%\right].$$

Moreover, we quantified the model performance using RMSE. Both metrics, RMSE and *ρ*, were used in the individualized beamlets experiment. Since MCsquare^36^ natively returns dose in the units of eV∕*g*∕proton, and Pastor-Serrano and Perkó^8^ to calculate RMSE used the 3D dose distribution scaled to Gy∕10^9^ particles, in order to provide fair comparison, the same scaling has been applied here.

MAPE for each 3D dose distribution was calculated as

(9) $$\mathrm{MAPE}\left(D_{\mathrm{crop}},\;{\overset{ˆ}{D}}_{\mathrm{crop}}\right)=\frac{1}{n_{v,\mathrm{eligible}}}\sum_{i=1}^{n_{v,\mathrm{eligible}}}\frac{|D_{{\mathrm{crop}}_{i}}-{\overset{ˆ}{D}}_{{\mathrm{crop}}_{i}}|}{|D_{{\mathrm{crop}}_{i}}|}\times 100\;\left[\%\right],$$

where *n*_*v*,eligible_ is the number of eligible voxels, that is, voxels where the reference dose is above 10% of the max dose in the considered reference 3D dose distribution calculated by MC. Although the gamma-index remains our primary benchmark for comparability with prior deep learning proton dose engines, MAPE is reported alongside it as a distance-independent error measure that is directly interpretable.

#### Individual beamlets range metrics

Because the distal fall-off is the most demanding region for dose prediction and the most relevant for proton range, we additionally compared ADoTA against MC using direct range metrics derived from the integrated depth-dose profile *I*(*z*) = ∑_*x*,*y*_*D*(*z*, *x*, *y*), with *z* the depth axis. Each *I*(*z*) is resampled from its native 2 mm spacing to Δ*z*∕20 by cubic interpolation. We define the Bragg-peak depth $R_{100}=\arg\;\max_{z}I\left(z\right)$ and the distal fall-off depth *R*_*f*_ as the deepest falling-edge depth *z*, where *I*(*z*) = *f* *I*(*R*_100_), evaluated at *f* = 0.8 and *f* = 0.2. We define Distal Fall-off Width (DFW), DFW = *R*_20_ − *R*_80_, quantifies the edge steepness. For each beamlet we compute the signed prediction-minus-MC (ADoTA-MC) differences Δ*R*_100_ (Bragg-peak location error), Δ*R*_80_ (range error), Δ*R*_20_ (distal-20% depth error), and ΔDFW (fall-off-width mismatch), and summarize them over the test set as the mean ± standard deviation.

#### Plan-level dose-volume metrics

At the plan level, we compared the dose-volume histograms (DVH) of the ADoTA and MC doses, reporting the signed target near-minimum dose error Δ*D*_98%_ and the mean absolute near-maximum dose error ∣Δ*D*_2%_∣ over the organs at risk (OAR), both as a percentage of the prescribed dose. We denote by *D*_*v*%_ the dose received by at least *v*% of a structure’s volume and by *D*_mean_ the mean dose over the structure. We additionally report absolute MC and ADoTA values and signed differences for the target’s *D*_98%_, coverage dose *D*_95%_, and near-maximum dose *D*_2%_, and signed differences in *D*_mean_ for each organ at risk.

Four distinct experiments were designed to test the performance and accuracy of the developed dose engine, using individual beamlets and full treatment plans.

• *Individual beamlets:* The speed and accuracy of predicting dose distributions of individual beamlets were evaluated on the beamlet-level test set defined in Section “Ground Truth Data Generation,” comprising 1400 thoracic and 2100 abdominal/pelvic beamlets.
• *Robustness to beamlet incidence angle:* Six patients were selected from the held-out test set described in Section “Ground Truth Data Generation”: 3 thoracic cases from LUNG-PET-CT-Dx and 3 abdominal/pelvic cases from Stage II Colorectal. The isocenter was defined at the image center. For each patient we drew 3 independent gantry angles $\alpha_{G}\sim U\left(0^{\circ },360^{\circ }\right)$ from a fixed seed, shared across both energies, and rotated the CT grid about the isocenter. Each rotated grid was evaluated at two energy levels, a low energy (*ϵ* = 102.6 MeV) and a high energy (*ϵ* = 135 MeV), over a uniform bixelgrid of size 18 × 18 spanning *θ*_*x*_, *θ*_*y*_ ∈ [ − 2^∘^, 2^∘^]. The resulting sweep comprises 6 × 2 × 3 × 324 = 11 664 beamlets, of which 11 531 satisfied the FD≥0.99 retention criterion (Equation (3)) and were evaluated. Ground truth was generated with MCsquare at 10^7^ primaries per beamlet. The same sweep was repeated on a homogeneous water phantom with a 50 mm air layer at the entrance, using one 18 × 18 grid per energy, as a geometrically invariant reference.
• *Full treatment plans:* A total of 8 treatment plans were constructed in the OpenTPS software,^35^ including 4 prostate cases and 4 lung cases. The primary objective of this experiment was to assess the robustness of the proposed method for full-plan dose calculation rather than to produce clinically deliverable plans. For the beamlet influence calculation *d*_*ij*_, MCsquare simulations were performed with 10 000 particles per beamlet, whereas the full-plan Monte Carlo reference dose was calculated using 10^8^ particles per beam. Because treatment plans were generated automatically by OpenTPS without constraining the spot energy selection, the thoracic plans included spot energies up to 176 MeV, exceeding the beamlet-level thoracic training range of 70 to 150 MeV; these cases therefore constitute a partial out-of-distribution evaluation for high-energy thoracic beamlets.
• *Beamlet direction-encoding cost: CPU and GPU-optimized comparison:* To quantify the computational consequence of replacing beamlet-aligned CT resampling with the analytical projection, we benchmarked two routes on every beamlet of a large prostate plan (5640 spots, two fields): (i) a DoTA-like route,^8^ in which each beamlet-centered CT subvolume is rotated to the beam’s-eye-view frame and the predicted dose is subsequently rotated back to the field-aligned CT patch, and (ii) the proposed ADoTA route, which constructs the analytical 3D Gaussian projection **Φ** directly on the shared field-aligned 2 × 2 × 2 mm^3^ grid. Each route was timed on both platforms. On the central processing unit (CPU), timing was single-threaded: the DoTA-like route used SciPy affine_transform (order 1, trilinear) for the rotation and resampling onto the beam’s-eye-view grid, while the ADoTA projection is analytic and needs no interpolation. On the GPU, both routes were timed across a batch-size sweep from 1 to 112, with the DoTA-like resampling performed by PyTorch grid_sample (bilinear, align_corners=True) and batched results reported both compute-only and including host-device data transfer. Every timing used perf_counter with CUDA synchronization around GPU work, one discarded warm-up call, and 5 repeats per unit, where a unit is one beamlet for the CPU baseline and one batch for the GPU-batched cells. The 5 repeats of a unit were first reduced to that unit’s median, which suppresses isolated scheduling spikes; the reported statistics are then means over units, that is over all 5640 beamlets on CPU or over all batches spanning them on GPU. Repeats and units were never pooled into a single sample. All timings used 32 bit floating point (single) precision on a single NVIDIA A40 GPU with a single CPU thread (48 cores available; SciPy’s affine_transform is single-threaded regardless). The plan-level comparison reruns both routes over all 5640 spots using the same batch size as the full-plan reconstruction pipeline of Section “Treatment plans,” with every processing stage other than the direction encoding itself held identical between routes. Both routes execute the ADoTA network; they differ only in how each beamlet’s input geometry is prepared, so what is timed is the preparatory geometric work rather than 2 different dose engines. Accordingly, the DoTA-like route is charged for the 2 resampling operations it requires and not for constructing an analytical projection channel, which a model taking a beam’s-eye-view aligned CT patch would not need.

## Results

### Individualized beamlets

Table 1 summarizes the agreement between ADoTA predictions and Monte Carlo ground truth for the 3500 beamlets of the held-out test set (Section “Ground Truth Data Generation”). Across all clinical criteria, ADoTA achieves consistently high Γ pass rates, with the abdominal/pelvic cohort showing near 100% performance (eg, 99.87% ± 0.23% for Γ(1%, 3 mm, 0.1%)) and the thoracic cohort passing rates remaining high but with larger variability (eg, 99.40% ± 0.86% under the same criterion). As expected, tightening the criteria reduces pass rates, most noticeably in the thoracic set (down to 97.86% ± 2.3% for Γ(1%, 2 mm, 3%)), indicating that the most challenging discrepancies occur under stricter spatial/dose tolerances. Complementary to the gamma analysis, the per-sample MAPE, evaluated at the 10% eligibility threshold defined in Section “Methods and Materials,” was 5.71% ± 1.91% for the thoracic cohort and 4.46% ± 1.05% for the abdominal/pelvic cohort, with a combined mean of 5.64% ± 1.89% across the 3500-beamlet test set. Lowering the eligibility threshold to 1% of the per-sample maximum reference dose raises MAPE to 12.62% ± 5.40% (thoracic) and 9.16% ± 2.36% (abdominal/pelvic), with a combined mean of 11.05% ± 4.63%, reflecting the well-known sensitivity of MAPE to low-dose voxels where small absolute differences translate into large relative errors. The range metrics show small cohort-level mean signed errors, but substantially larger standard deviations in the thoracic cohort than in the abdominal/pelvic cohort, particularly for Δ*R*_80_ and ΔDFW. Because positive and negative errors partially cancel in a signed mean, absolute errors, percentiles, and threshold-exceedance fractions for the same beamlets are reported in Appendix A.2, Table A2. For the clinical range metric Δ*R*_80_, the mean absolute error (MAE) was 1.66 mm in the thoracic cohort and 0.57 mm in the abdominal/pelvic cohort, and the 95th percentile of ∣Δ*R*_80_∣ was 4.90 and 1.86 mm, respectively. 10.0% of thoracic beamlets exceeded a 3 mm absolute range error and 5.4% exceeded 5 mm, compared with none of the abdominal/pelvic beamlets at either threshold. Further complementary error metrics (relative dose error *ρ*, RMSE, and MAPE) are provided in Appendix (Section A.1).

Figure 3a and b provide representative, mean-performing beamlets selected by the per-sample global Γ(2%, 2 mm, 10%) pass rate. The key qualitative observations are: (i) close spatial agreement of the high-dose region in axial/sagittal views, (ii) preservation of the overall depth–dose trend and approximate Bragg peak location in the IDD profiles, despite visible discrepancies in peak magnitude for the thoracic example, and (iii) limited reproduction of the sharp distal dose shape in heterogeneous tissue, leading to a smoothed Bragg peak compared with MC. Best- and worst-performing cases for each cohort are reported in the Appendix (Section A.7; Figures A15, A16, A17, A18).

**Figure 3:**
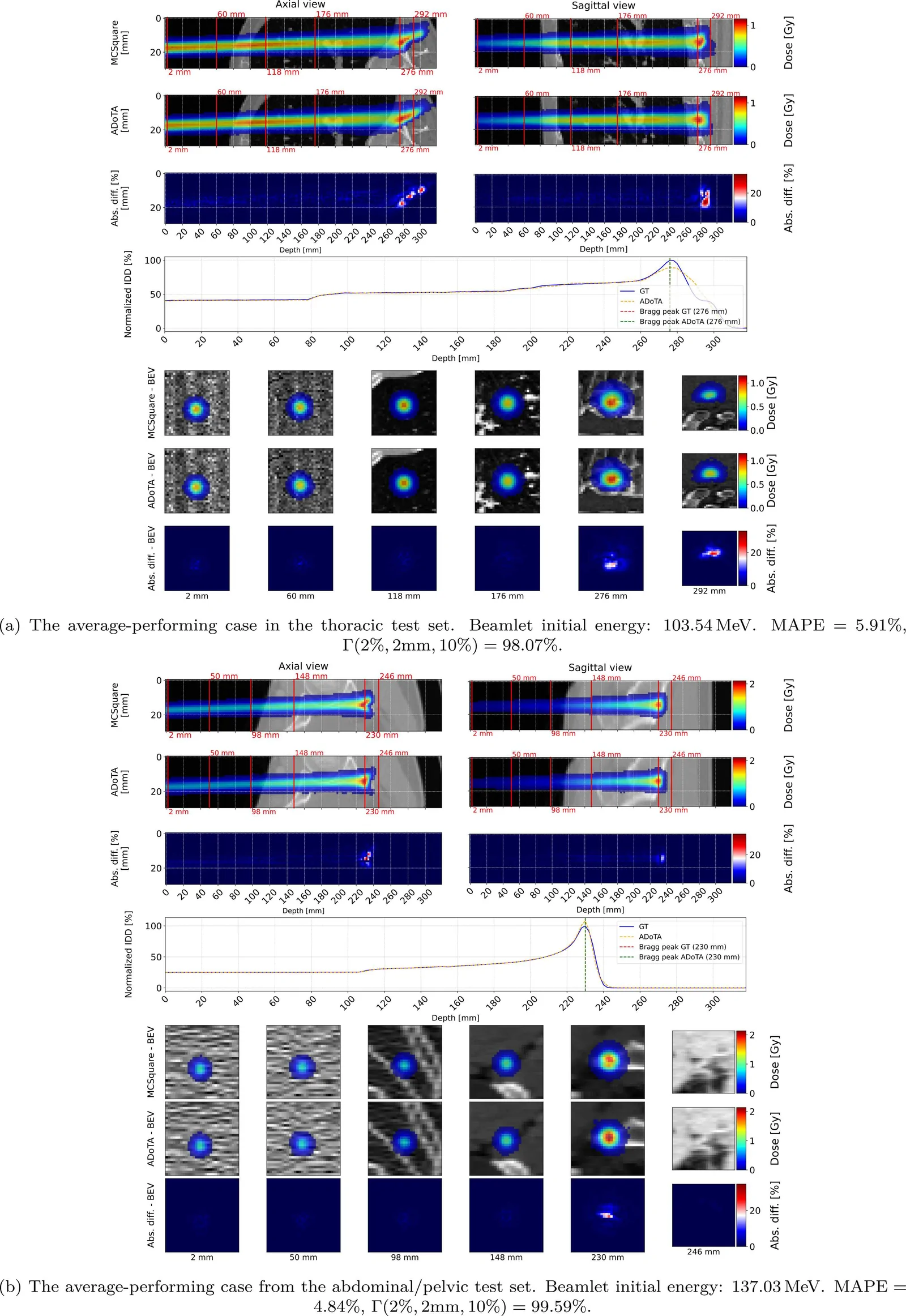
The first 3 rows display the 3D dose distributions calculated by MCsquare (first row) and ADoTA (second row), along with the absolute percentage difference between them (third row). The axial and sagittal planes are centered on the voxel containing the Bragg peak. The fourth row presents the normalized IDD profiles for both the MCsquare and ADoTA distributions. The final 3 rows show the beam’s eye view at selected depths, as indicated by the red markers in the upper rows. Only voxels receiving a dose above 10 % of the maximum deposited dose are included in the MAPE calculation.

Depth-resolved performance is reported in the Appendix (Section A.4), where we analyze the mean Γ pass rate computed per lateral depth slice as a function of beamlet penetration depth (Figure A2a; Γ(1%, 3 mm, 0.1%)), together with the fraction of test samples exhibiting non-zero dose deposition at each depth (Figure A2b), which decreases with depth due to the finite proton range.

Aggregated over the 11 531 beamlets of the incidence-angle sweep (Section “Model evaluation”), ADoTA reproduces the Monte Carlo reference with a dose passing rate of Γ(2%, 2 mm, 10%) = 96.05% ± 2.13%. Performance separates by anatomy rather than by beam configuration: the abdominal/pelvic cases reach 96.88% ± 1.44% against 95.20% ± 2.38% for the thoracic cases, consistent with the greater tissue heterogeneity of the lung. Pooled over both cohorts, the 2 energies separate the pass rate less than the 2 anatomical sites do (96.11% at 102.6 MeV against 95.98% at 135 MeV); this comparison covers 2 energies in the middle of the trained range over 6 geometries and does not characterize the energy dependence of the model in general. Across the 18 × 18 grid of scanning angles the pass rate shows no systematic trend in *θ*_*x*_ or *θ*_*y*_, and the variation that does occur follows the anatomy traversed by the beamlet rather than its incidence angle (Appendix A.6); the 3 gantry angles drawn per patient agree to within approximately one percentage point (Appendix A.5). Mean pass rates per scanning angle, averaged over the 9 sweeps of each anatomical site (3 patients, 3 gantry angles each), are shown in Figure 4a and b for the low energy beam and in Figure 4c and d for the high energy beam; the corresponding validation on the water phantom, with Γ(1%, 3 mm, 0.1%) criteria, is shown in Figure 4e and f. Additional energies and gantry angles are reported in Appendix A.5.

**Figure 4:**
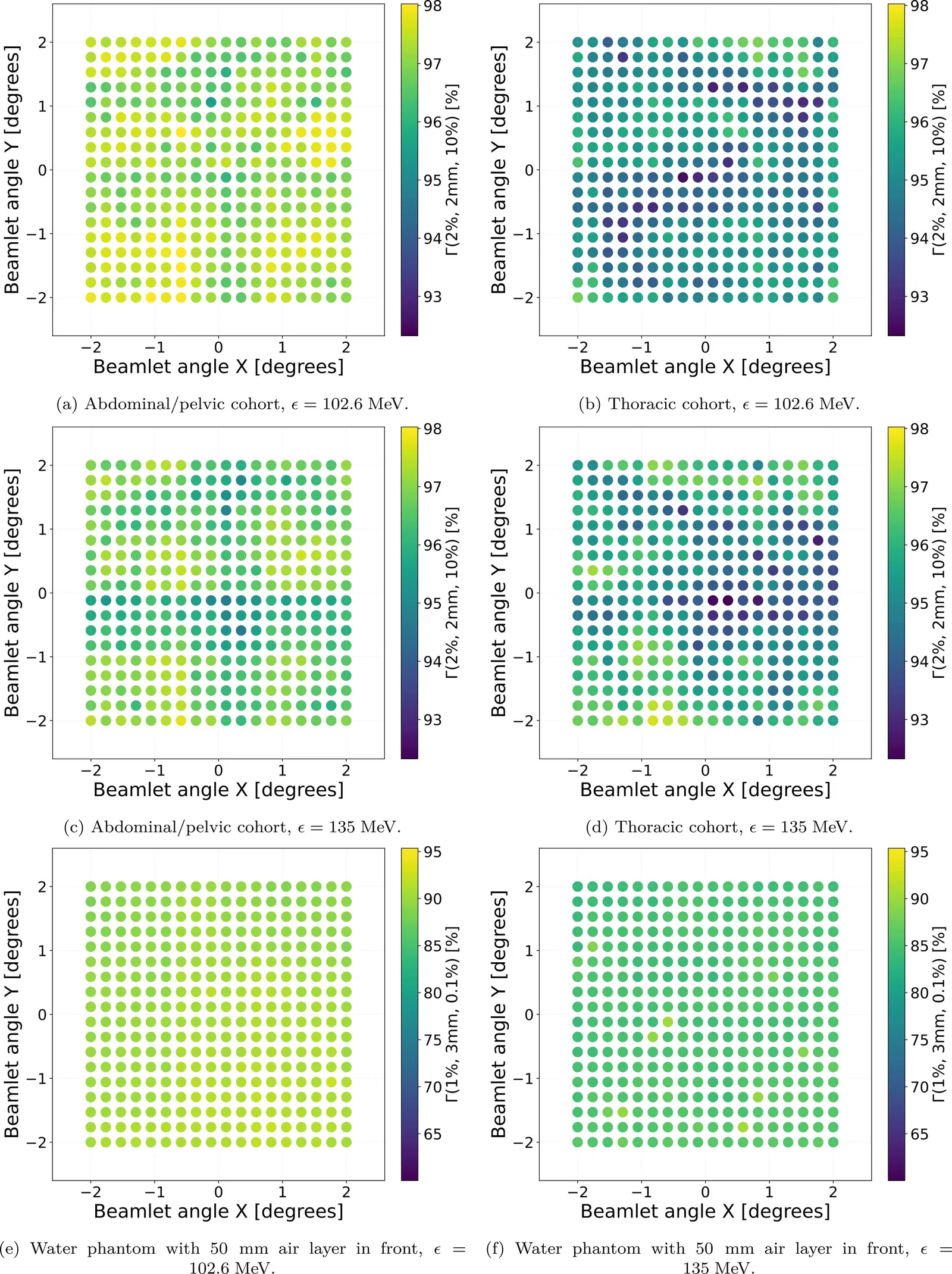
ADoTA geometric robustness evaluation across varying beamlet incidence angles on 3 abdominal/pelvic CTs, 3 thoracic CTs, and one homogeneous water phantom (Section “Model evaluation). (a)-(d) Patient cohorts: each cell shows the Γ(2%, 2 mm, 10%) pass rate at that scanning angle (*θ*_*x*_, *θ*_*y*_), averaged over the 9 sweeps of the corresponding anatomical site (3 patients with 3 gantry angles each, up to 2916 individually simulated beamlets per panel); these 4 panels share a common color scale, so colors are directly comparable between anatomical sites and energies. (e)-(f) Homogeneous water phantom with a 50 mm air layer at the entrance, evaluated under the Γ(1%, 3 mm, 0.1%) criterion; each panel represents 324 beamlets. The 36 individual per-patient, per-gantry panels are reported in Appendix A.5.

### Ablation of the IDD loss and residual connections

Table A3 summarizes the validation-set ablation results for the IDD loss, the encoder–decoder convolutional residual connections, and the analytical projection channel. The full ADoTA configuration achieved the lowest MAPE (6.02% ± 4.67%) and the smallest spread in all reported range metrics, with Δ*R*_80_ = −0.232 ± 5.665 mm, Δ*R*_20_ = 0.133 ± 4.372 mm, and ΔDFW = 0.365 ± 5.394 mm. Removing the IDD term increased MAPE to 9.17% ± 5.46% and substantially broadened the range-error distributions, most notably for Δ*R*_80_ (0.779 ± 8.681 mm) and ΔDFW −0.476 ± 10.206 mm). Removing the convolutional residual connections caused a smaller but consistent degradation, increasing MAPE to 7.47% ± 4.56% and yielding intermediate range-error variability. Replacing the energy-dependent projection **Φ** with the constant-width centerline projection **Φ**^*E*^ increased MAPE only slightly, to 6.28% ± 4.57%, close to the full configuration and well below the degradation caused by removing either the IDD term or the residual connections. The range-metric picture is mixed: **Φ**^*E*^ showed a smaller spread than the full configuration on all 3 range metrics (Δ*R*_80_ = −0.039 ± 4.660 mm, Δ*R*_20_ = −0.700 ± 3.556 mm, ΔDFW = −0.675 ± 4.895 mm), but with a larger mean bias on Δ*R*_20_ and ΔDFW than the full configuration. Replacing **Φ**^*E*^ with its hard, one-voxel-wide limit **Φ**^*F*^ (binary centerline, no Gaussian falloff) degraded MAPE further to 8.85% ± 4.81%, with Δ*R*_80_ = −0.219 ± 4.514 mm, Δ*R*_20_ = 0.274 ± 3.130 mm, and ΔDFW = 0.590 ± 4.391 mm: a larger MAPE penalty than **Φ**^*E*^, though still well below removing the IDD term. These results indicate that the IDD term is the dominant contributor to range fidelity, the residual connections provide an additional architectural benefit, and most of the projection’s value derives from encoding the beam direction and entrance point rather than the energy-dependent spot width, though its effect on the distal range metrics specifically is not uniformly positive.

### Treatment plans

As a proof of concept that individual beamlet predictions compose into a coherent full-plan dose, we employed ADoTA to compute the 3-dimensional dose distribution for full treatment plans. The details and performance of each plan are presented in Table 2a. Gamma pass rates were consistently high across all 8 cases, while the target near-minimum dose error Δ*D*_98%_ differed systematically by anatomical site, as discussed in Section “Discussion.” The achieved performance is later compared in Table 2b to the model performance reported in the work of Pastor-Serrano and Perkó^8^ and Pang et al.^29^

The full-plan reconstruction pipeline was implemented to avoid writing and reading intermediate beamlet files. For each gantry angle, the CT was first resampled once to the ADoTA-native 2 × 2 × 2 mm^3^ grid and rotated by the gantry angle. Beamlet subvolumes were then cropped directly from this shared grid, grouped first by gantry angle and subsequently processed in batches of 120 beamlets. In this full-plan ADoTA implementation, the analytical beamlet shape projection **Φ** and network inference were evaluated on the GPU, after which the predicted beamlet doses were multiplied by their spot weights and accumulated in memory into an ADoTA dose-scoring grid copied from the CT grid. Only the final accumulated full-plan dose was written to disk, avoiding intermediate input/output for individual beamlets. To improve throughput, GPU operations were performed using torch.autocast mixed precision, converting 32 bit floating point operations (FP32) to 16 bit floating point operations (FP16) where supported; in our implementation this reduced runtime by approximately 30% relative to FP32 execution.

In the Appendix, Figure A19 illustrates representative full-plan dose distributions, and Figure A20, Figure A21 present the DVHs for all 8 plans.

### Runtime

Table A4 reports the network inference times, which depend strongly on the batch size. A beamlet processed on its own required 14.1 ms, whereas batched processing reduced the effective per-beamlet time to 3.34 ± 0.08 ms at a batch size of 56 and to 3.14 ± 0.31 ms at a batch size of 200. Full-plan reconstruction evaluates thousands of independent beamlets and therefore operates in the batched regime, so the batched value is the one relevant for plan-level runtime, while the single-beamlet value bounds the latency of an isolated prediction. Appendix A.8 provides the complete breakdown.

In a single-threaded CPU version of the benchmark described in Section “Methods and Materials,” the ADoTA analytical projection required 9.06 ms per beamlet against 48.10 ms for the 2 DoTA-like resampling operations; this unoptimized comparison is retained only as CPU context (Appendix A.9). With both routes instead optimized on GPU and batched in single precision (Appendix A.9, Table A5), the analytical projection was 3.1–4.0× cheaper on compute and 6.1–6.9× cheaper, including host-device transfer, than batched local beam’s-eye-view sampling at batch sizes of 32 to 56, where the fixed per-batch overhead is spread over enough beamlets to become negligible, per beamlet. Applied to the same plan, run end to end on all 5640 spots (Table A6), this reduced the plan’s total direction-encoding cost from 25.46 to 2.64 seconds (9.6×); since the model forward pass and remaining geometry stages are shared and near-identical between routes, the plan’s total compute time was reduced by 1.48× (compute-only, excluding disk I/O and data loading), from 63.57 to 43.07 seconds.

## Discussion

In this work, we address a central limitation of existing deep-learning-based proton dose engines, namely the requirement to resample a local beam’s-eye-view CT patch for every beamlet in order to enforce perpendicular alignment between the beam direction and the voxel grid. We replace this per-beamlet resampling with an additional input channel encoding an analytically computed Gaussian beamlet shape projection so that a single CT rotation and resampling per irradiation field remains (Section “Ground Truth Data Generation”).

The computational advantage of this substitution is structural rather than an artifact of implementation quality. In a GPU-optimized benchmark on the same plan (Appendix A.9), constructing the analytical projection remained 3.1-4.0× cheaper per beamlet than per-beamlet CT and dose-patch rotation on compute alone, 6.1-6.9× cheaper once host-device transfer is included, and reduced the plan’s total compute time by 1.48×. The advantage that persists under transfer follows from what each route must move: local beam’s-eye-view sampling transfers a CT subvolume to the device and the resampled result back for every beamlet, whereas the analytical projection is built on the device from a few per-beamlet scalars, namely the entrance point, the steering angles, and the energy-dependent spot width (Table A5).

Regarding geometric generalization, the model maintained high predictive accuracy at every beamlet orientation tested, supporting the claim that the strict orthogonality between the proton ray and the voxel grid required by prior methods^8^, ^29^, ^30^ is not a prerequisite for accurate prediction. By explicitly encoding the incident trajectory through the analytical beamlet shape projection, the network maps varying scanning deflections onto a single field-aligned grid, which is what removes the beamlet-specific local beam’s-eye-view resampling of prior approaches. The tested scope, 8 patient geometries at 5 energies between 80 and 140 MeV plus one water phantom, including a 6-patient sweep with 3 gantry angles per patient (11 531 beamlets; Figure 4a-f; Appendix A.5), is an extended sensitivity analysis rather than a population-level generalization study, and does not establish invariance to beam orientation across anatomical sites or the full clinical energy range.

The variation that does occur within a single angular grid panel of Figure 4a-f follows the anatomy the beamlet traverses rather than the incidence angle. Along the anti-diagonal of the thoracic 80 MeV panel of Figure A8d, the pass rate tracks the lung fraction of the beamlet path and steps where that path stops crossing lung, while the incidence angle varies smoothly throughout (Section A.6).

It is important to note the scope of this generalization. The orientation sweep validates that the beam-optics parameters *explicitly* carried by **Φ**, namely nominal field direction, off-axis spot direction, virtual-source geometry, and initial-energy-dependent in-air spot size (Section “Ground Truth Data Generation”), generalize consistently across gantry angles and lateral scanning deflections. The remaining beam-optics components, namely finite angular divergence at the nozzle, multiple Coulomb scattering, nuclear interactions, range straggling, and heterogeneity-driven dose perturbations, are *implicitly* learned from Monte Carlo labels generated with a single BDL, and their generalization to delivery systems with materially different beam optics has not yet been demonstrated; a cross-machine experiment isolating this dependency is planned as future work.

The value of the analytical projection is best tested against a simpler direction encoding rather than by removing it outright, since some encoding of beam direction is required once per-beamlet CT rotation is removed. Two such encodings were trained and are reported in Table A3: a constant-width tube on the same centerline, which withholds only the energy-dependent spot width, and its zero-width limit, a binary centerline map, which withholds the lateral extent and the sub-voxel position of the beam axis as well. Withholding the width alone cost little; withholding the lateral extent as well cost substantially more. The value of the projection therefore lies chiefly in supplying explicit directional geometry on a field-aligned grid, while the energy-dependent width and the Gaussian falloff shape are secondary refinements, and simpler direction encodings remain viable but less accurate design choices.

While the geometric performance is uniform, prediction fidelity exhibits specific dependencies on the physical beam range. We observed a reduction in agreement in the distal 25% of the calculation volume depth (Section A.4, Figure A2a). This trend arises from the combined effect of reduced representation of deep penetration regions in the training data and the increased physical complexity of dose deposition near the Bragg peak, where steep dose gradients and range straggling dominate. First, as illustrated in Section A.4, Figure A2b, the training distribution exhibits an inherent imbalance with respect to penetration depth. While dose deposition occurs in the entrance region for every beamlet regardless of energy, the maximum penetration depth is a direct function of the initial beam energy. Second, this data sparsity coincides with the region of highest physical complexity. This aligns with findings by Pastor-Serrano and Perkó,^8^ who partitioned the beam path into 4 equal-depth sections and showed that the final section, corresponding to the Bragg peak and distal fall-off of the highest energy beams, consistently exhibits the highest relative error. This confirms that the coupling of specific data sparsity and gradient complexity renders the distal edge the dominant challenge for deep-learning-based dose engines. Consistent with this, the IDD ablation (Table A3) shows that explicitly penalizing depth–dose discrepancies improves overall agreement, supporting the inclusion of the IDD term in the training objective.

The direct range metrics localize this limitation more precisely than the gamma pass rates alone. The mean signed errors are small in both cohorts, so the model carries no large systematic range shift; what separates the cohorts is dispersion, with thoracic standard deviations of 3.5–5.8 mm across Δ*R*_80_, Δ*R*_20_, and ΔDFW against sub-millimeter values for abdominal/pelvic beamlets (Table 1; Appendix A.2, Table A2). This follows from the traversed anatomy: thoracic beamlets cross low-density lung, ribs, and air–tissue boundaries, where a small spatial or density error displaces the distal crossing points by millimeters even when the 3-dimensional gamma agreement remains high, whereas the more homogeneous abdominal/pelvic paths yield a stable distal fall-off. ADoTA therefore reproduces the average Bragg-peak position and distal-edge shape, but isolated thoracic beamlets remain vulnerable to heterogeneity-driven range errors, so range-sensitive use near critical structures requires explicit distal-edge evaluation rather than gamma pass rates alone.

The target near-minimum dose error Δ*D*_98%_ is tied to anatomical site rather than to beam energy or plan complexity. Across the 8 evaluated plans, ∣Δ*D*_98%_∣ falls into 2 tight, non-overlapping bands: 1.8–2.5% for the 4 prostate plans and 4.2% to 5.9% for the 4 thoracic plans (Table 2a). The same cohort separation appears in the beamlet-level range dispersion described above (Table 1).

A dispersion of the per-beamlet range error is one candidate for this deficit. *D*_98%_ is fixed by the coldest 2% of target voxels, so a beamlet that stops short leaves a cold region at the distal target margin that a neighboring beamlet stopping long does not fill. Symmetric, unbiased range errors would therefore lower *D*_98%_ one-sidedly, which is consistent with the small signed mean range errors in both cohorts and the larger thoracic dispersion. This link is not tested here; establishing it would require results that tie individual beamlet range errors to the resulting plan dose directly. It would also account only for the near-minimum dose and not for the broader target underestimation seen in several plans: the thoracic *D*_95%_ is underestimated by 3.8% to 5.2% of the prescribed dose, and Prostate 1 underestimates the target *D*_2%_ by 4.0% (Appendix A.10, Table A7). The origin of that broader deficit remains open.

Two further candidate explanations were tested directly and are not supported. Out-of-distribution beam energy does not account for the pattern: Thoracic 2 never exceeds the beamlet-level thoracic training range of 150 MeV (maximum 137 MeV) yet shows Δ*D*_98%_ = −5.4%, within the same band as the partially out-of-distribution thoracic plans (Appendix A.12). Dense-spot accumulation, whereby a local bias for a given tissue configuration would be reproduced across many nearby beamlets rather than averaging out,^48^ predicts the opposite of what is observed: Thoracic 4 has the most spots of any evaluated plan (5019) but the smallest thoracic bias (−4.2%).

The organ-at-risk errors are directional and structure-specific rather than uniformly negative (Appendix A.10, Table A7). Across all 4 prostate plans, Bladder *D*_mean_ is consistently over-estimated (+0.6 to +1.5%) while Rectum is consistently under-estimated (−1.8 to −2.4%); the cause of this directional split is not established here. Thoracic 3 is a partial exception to the overall pattern, with its target *D*_2%_ over- rather than under-estimated +8.8%), indicating that the bias is not uniformly directional at the high-dose end of every plan.

Inference precision is not a contributor. A dedicated FP16-versus-FP32 comparison on one representative prostate and one representative thoracic plan (Appendix A.11, Table A8) shifted every gamma criterion by at most 0.025 percentage points and left RMSE and the relative dose error *ρ* unchanged to within 0.01 in their respective units. These shifts are far smaller than the target-dose biases in Table 2a, which reach several percent of prescription, so the mixed-precision inference used throughout does not explain them.

A high plan-level gamma pass rate is not evidence of comparably accurate target coverage. Mean Γ(2%, 2 mm, 10%) was 95.1% for the lung cohort and 96.1% for the prostate cohort, yet this aggregate metric does not capture the systematic *D*_98%_ underestimation reported in Table 2a. The thoracic bias in particular should be resolved before the method is considered for clinical use.

We emphasize, however, that the scope of this work is the beamlet-level dose engine itself. As motivated in the Introduction, the dose-influence matrix *d*_*ij*_ that underpins intensity-modulated proton therapy optimization is assembled column by column from individual beamlet dose distributions, and our objective is to provide the accurate and fast per-beamlet engine that populates it. The treatment-plan results are therefore reported as a proof of concept that these beamlet predictions compose into a coherent full-plan dose, rather than as a clinical validation. We nevertheless include a limited plan-level DVH assessment: target near-minimum dose differences (Δ*D*_98%_) and OAR near-maximum dose differences (∣Δ*D*_2%_∣), expressed as percentages of the prescribed dose, are reported in Table 2a; representative full-plan dose distributions are shown in Figures A19a and b, and DVHs for all 8 plans are shown in Figure A20, Figure A21. A dedicated hot-spot analysis and broader organ- and site-specific clinical validation, including distal-edge errors near critical structures and tissue interfaces, remain beyond the present scope.

From a runtime perspective, the full-plan reconstruction pipeline benefits from batching independent beamlet dose predictions. This enables sub-minute reconstruction even for large scanned-spot plans: the largest evaluated case, Prostate 2, contained 9273 spots and was reconstructed in 52.11 seconds (Table 2a). The analytical projection **Φ** is constructed on the GPU alongside the network inference, so this step no longer acts as a CPU bottleneck. The remaining CPU-side operations, namely the per-field CT rotations and the cropping and reassembly of beamlet subvolumes, are vectorizable and may benefit from further GPU implementation;^49^ however, this optimization was not evaluated in the present work.

Several limitations must also be acknowledged. First, the model was trained exclusively on isotropic 2 × 2 × 2 mm^3^ grids. Although the convolutional architecture itself does not require this resolution at inference, the learned feature representations are implicitly tied to this sampling scale. Accordingly, the extent to which the model generalizes across voxel spacings remains unknown and should be established explicitly in future work. Second, although the inclusion of beam projection information may provide a mechanism for extending the framework across different machine-specific beam optics, this capability was not evaluated in the present study. Both training and evaluation were performed using a single beam model, and thus generalization across delivery systems remains a hypothesis rather than a demonstrated property of the method. Finally, the fixed crop depth (320 mm) prevents prediction for very deep-seated targets. Future iterations must therefore incorporate dynamic cropping or autoregressive depth extension to accommodate arbitrary path lengths. Addressing these limitations will be essential for establishing a generalizable and clinically deployable deep learning dose engine.

## Conclusion

This study introduces a beamlet angle-aware deep learning framework, built on a Transformer encoder architecture, that achieves near Monte Carlo accuracy at the beamlet level across the trained energy ranges. The approach removes the perpendicularity requirement between proton ray directions and the CT voxel grid so that the geometric preparation is reduced from one local beam’s-eye-view resampling per beamlet to one CT transformation per irradiation field. Full-plan target coverage remained systematically biased, particularly in thoracic anatomy, and this bias should be resolved before the method is considered for clinical deployment. The method nonetheless exhibits strong potential for integration into time-constrained online adaptive proton therapy workflows and for constructing the dose-influence matrix *d*_*ij*_ within robust optimization frameworks, where multiple spot-selection scenarios must be evaluated efficiently to ensure conformal and uncertainty-tolerant dose delivery.

## Funding

This work is supported by the TU Delft AI Labs program.

## Declaration of Conflicts of Interest

Zoltán Perkó is an associate professor at TU Delft and is employed as a Senior Applied Scientist at Radformation Inc. His industry employment is unrelated to the submitted work. The remaining authors have no conflicts of interest to declare.

## Declaration of Generative AI and AI-Assisted Technologies in the Manuscript Preparation Process

During the preparation of this work, the author(s) used GPT-5.2 (OpenAI) and Gemini 3 Pro (Google) to correct grammar and improve clarity. After using this tool/service, the author(s) reviewed and edited the content as needed and take(s) full responsibility for the content of the published article.

## CRediT authorship contribution statement

**Mikołaj Stryja:** Data curation, Formal analysis, Investigation, Methodology, Software, Validation, Visualization, Writing – original draft. **Danny Lathouwers:** Conceptualization, Formal analysis, Methodology, Project administration, Supervision, Resources, Writing – review & editing. **Zoltán Perkó:** Conceptualization, Formal analysis, Funding acquisition, Methodology, Project administration, Supervision, Resources, Writing – review & editing.

## Figures and Tables

**Table A1:** Performance of ADoTA compared to the original DoTA algorithm introduced by Pastor-Serrano and Perkó.^8^

Model	Anatomical site		Relative Dose Error [%]			Root Mean Square Error [Gy/10^9^]		MAPE [%]
		Mean±std	Min	Max	Mean±std	Min	Max	Mean±std
DoTA	Combined	0.126±0.109	0.025	1.258	0.083±0.041	0.024	0.277	–
ADoTA	Combined	0.044±0.035	0.0073	0.388	0.0104±0.0043	0.0042	0.0308	5.64±1.89
	Lungs	0.0738±0.0437	0.009	0.388	0.013±0.005	0.0042	0.0308	5.71±1.91
	Abdominal/Pelvic	0.0319±0.043	0.00730	0.2231	0.0087±0.0027	0.0049	0.022	4.46±1.05

RMSE is calculated based on the 3D dose distribution deposited by the 10^9^ particles converted to Gray units. MAPE is reported at the 10% eligibility threshold defined in Section “Methods and Materials.”

**Figure A1:**
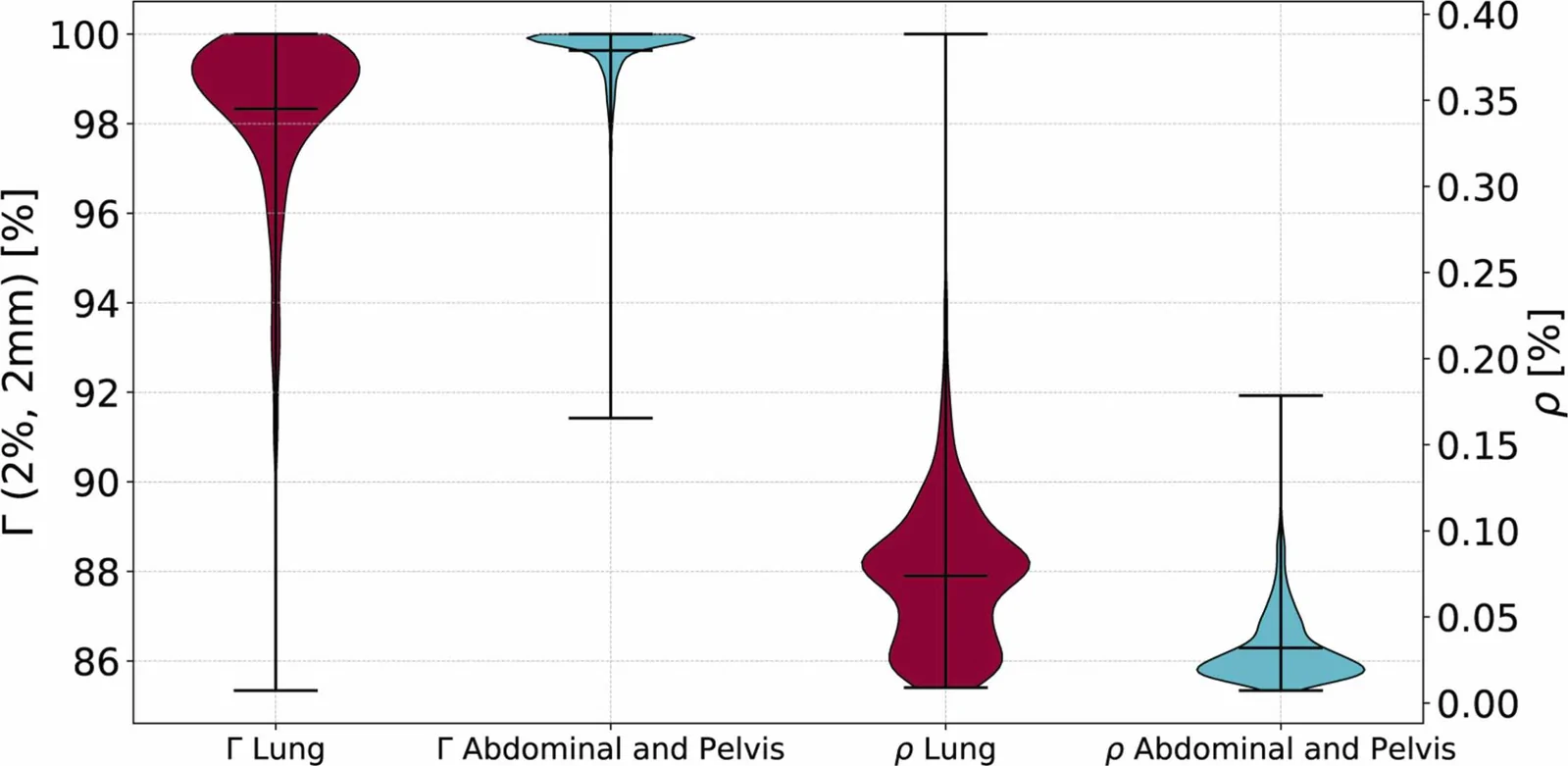
Distribution of gamma pass rate Γ(2%, 2 mm, 10%) and relative dose error *ρ* on the lung and abdominal/pelvic beamlet test datasets, comprising 1400 lung and 2100 abdominal/pelvic beamlets (3500 beamlets in total, drawn from 50 test patients; Section “Methods and Materials”). For each distribution, markers indicate the minimum, mean, and maximum values across the corresponding test samples.

**Table A2:** Distribution of beamlet-level range errors on the 3500-beamlet test set (Section “Ground Truth Data Generation”), complementing the signed means reported in Table 1.

			Signed error [mm]		Absolute error [mm]			Beamlets exceeding [%]	
Metric	MAE [mm]	*p*_5_	*p*_50_	*p*_95_	*p*_95_	max	2 mm	3 mm	5 mm
				*Combined (3500 beamlets)*					
Δ*R*_100_	1.05	−3.11	−0.45	0.81	3.81	14.40	11.7	7.1	2.5
Δ*R*_80_	1.16	−1.92	−0.30	2.72	3.11	56.14	9.6	5.4	2.9
Δ*R*_20_	1.14	−2.37	−0.20	2.15	5.51	19.93	12.1	8.8	5.4
ΔDFW	1.44	−3.96	0.12	2.37	6.03	48.84	15.4	11.3	6.3
				*Thoracic (1400 beamlets)*					
Δ*R*_100_	1.41	−4.37	−0.50	1.16	4.46	14.40	18.5	11.5	4.6
Δ*R*_80_	1.66	−2.06	−0.30	4.38	4.90	56.14	15.4	10.0	5.4
Δ*R*_20_	1.72	−5.74	−0.21	3.83	7.64	19.93	22.3	16.2	10.0
ΔDFW	2.30	−6.81	0.11	3.67	9.25	48.84	26.2	18.5	11.5
				*Abdominal/pelvic (2100 beamlets)*					
Δ*R*_100_	0.63	−1.90	−0.40	0.30	1.96	4.00	3.6	1.8	0.0
Δ*R*_80_	0.57	−1.26	−0.29	0.89	1.86	2.96	2.7	0.0	0.0
Δ*R*_20_	0.46	−1.15	−0.19	0.64	1.24	1.96	0.0	0.0	0.0
ΔDFW	0.43	−0.59	0.14	0.93	1.53	3.95	2.7	2.7	0.0

Signed percentiles show the 2-sided spread; absolute-error percentiles and threshold-exceedance fractions quantify the magnitude of individual errors irrespective of sign. All values in millimeters except the exceedance fractions.

**Table A3:** Ablation study on the validation set (14 029 beamlets); configurations described in Section A.3.

Configuration	MAPE [%]	Δ*R*_80_ [mm]	Δ*R*_20_ [mm]	ΔDFW [mm]
MSE + IDD (ADoTA)	**6.02**±**4.67**	−**0.232**±**5.665**	**0.133**±**4.372**	**0.365**±**5.394**
MSE only	9.17±5.46	0.779±8.681	0.303±7.538	−0.476±10.206
MSE + IDD, w/o conv. residual	7.47±4.56	−0.532±6.425	−0.262±4.463	0.270±6.379
MSE + IDD, constant-width centerline **Φ**^E*^	6.28±4.57	−0.039±4.660	−0.700±3.556	−0.675±4.895
MSE + IDD, binary centerline **Φ**^F*^	8.85±4.81	−0.219±4.514	0.274±3.130	0.590±4.391

Values are mean ± standard deviation across beamlets. Lower is better.

**Figure A2:**
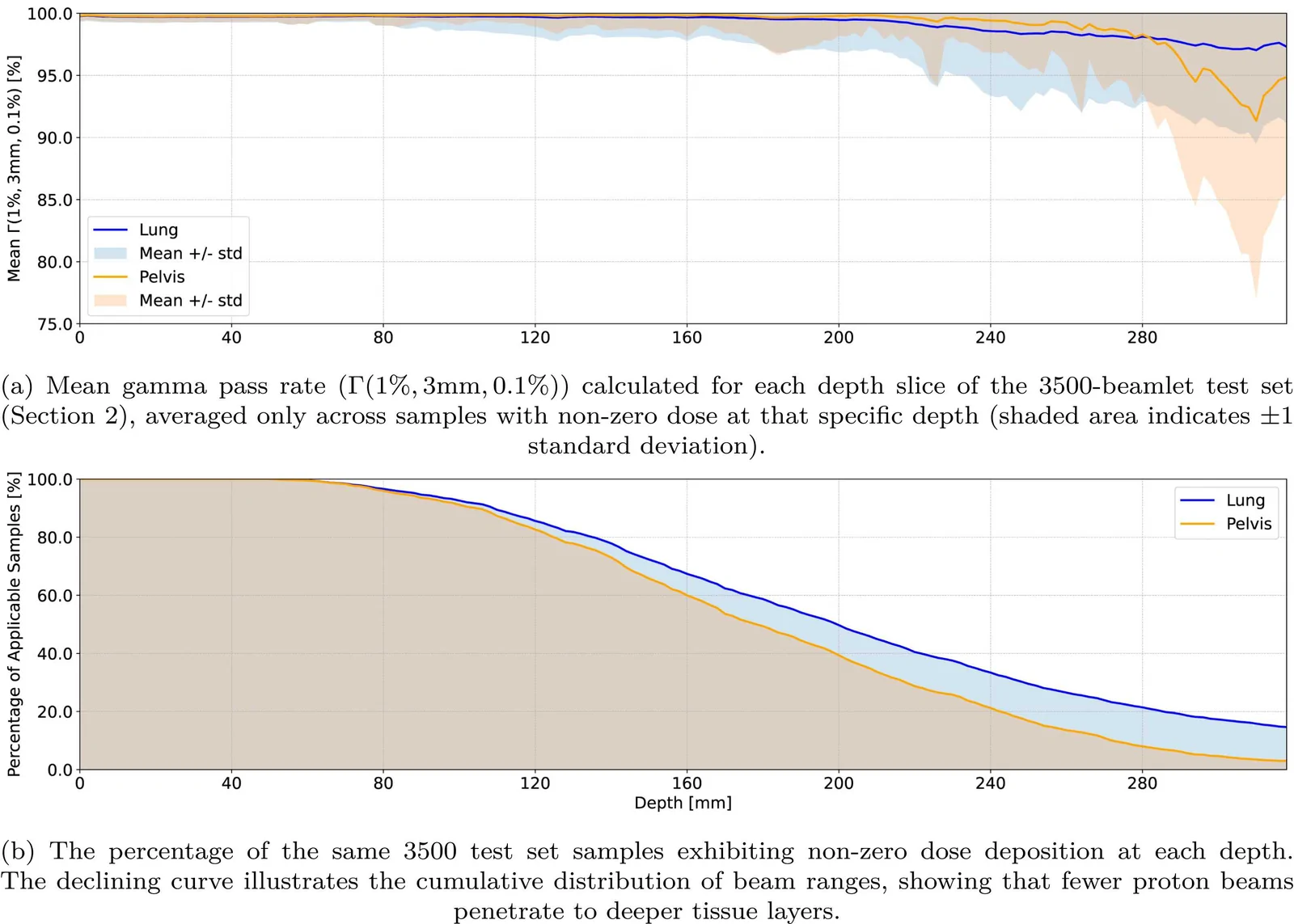
Depth-wise model performance analysis.

**Figure A3:**
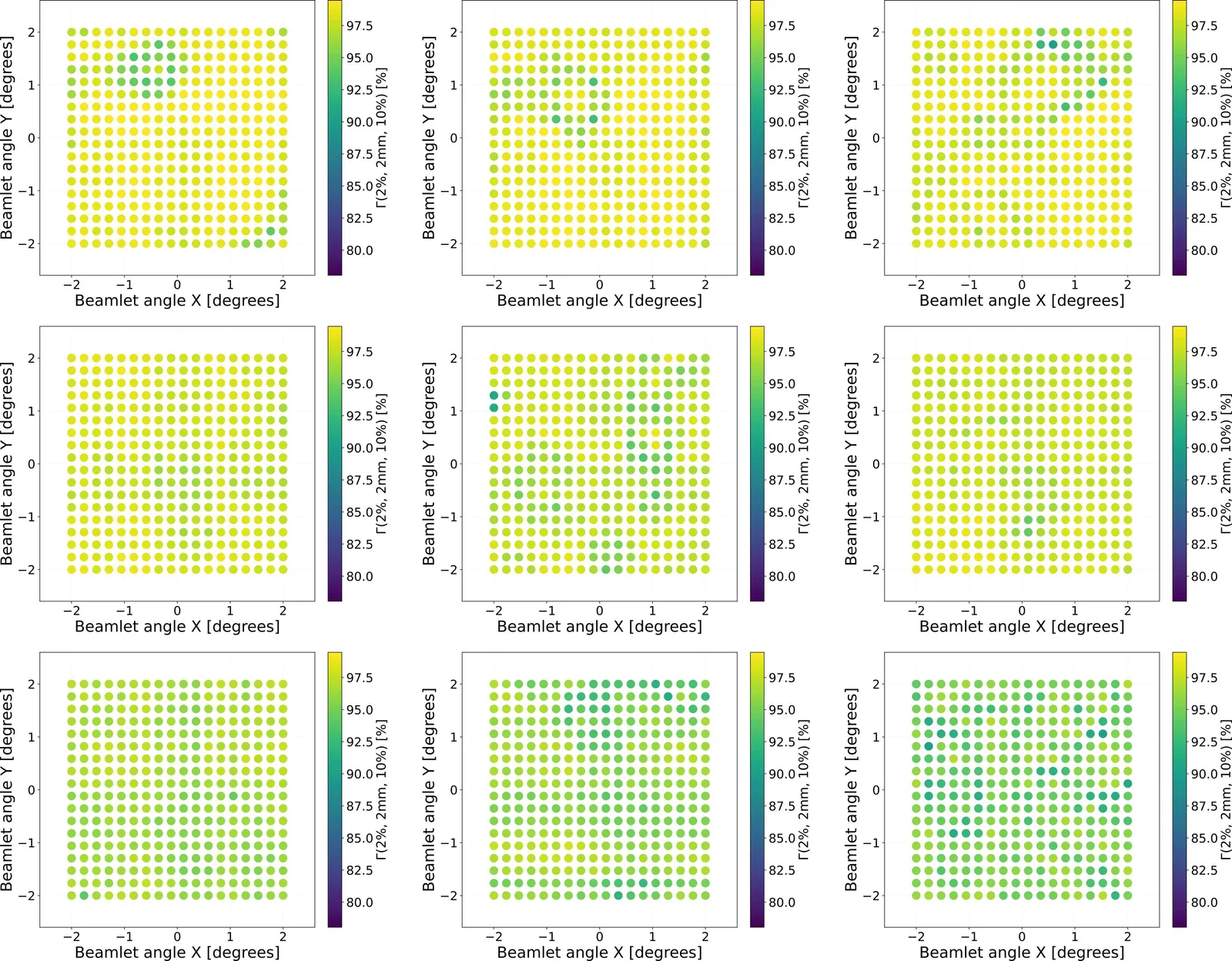
Per-patient, per-gantry Γ(2%, 2 mm, 10%) pass rates of the abdominal/pelvic cohort at *ϵ* = 102.6 MeV, underlying the aggregate panel of Figure 4a. Each panel covers the 18 × 18 grid of scanning angles *θ*_*x*_, *θ*_*y*_ ∈ [ −2^∘^,2^∘^] (324 beamlets). Rows, top to bottom: patients StageII-Colorectal CT-222, CT-225, and CT-230; within each row, that patient’s 3 gantry angles in ascending order (CT-222: 93.4^∘^, 114.6^∘^, 197.8^∘^; CT-225: 2.4^∘^, 144.0^∘^, 340.1^∘^; CT-230: 27.6^∘^, 82.0^∘^, 188.4^∘^). All panels of Figure A3, Figure A4, Figure A5, Figure A6 share one common color scale.

**Figure A4:**
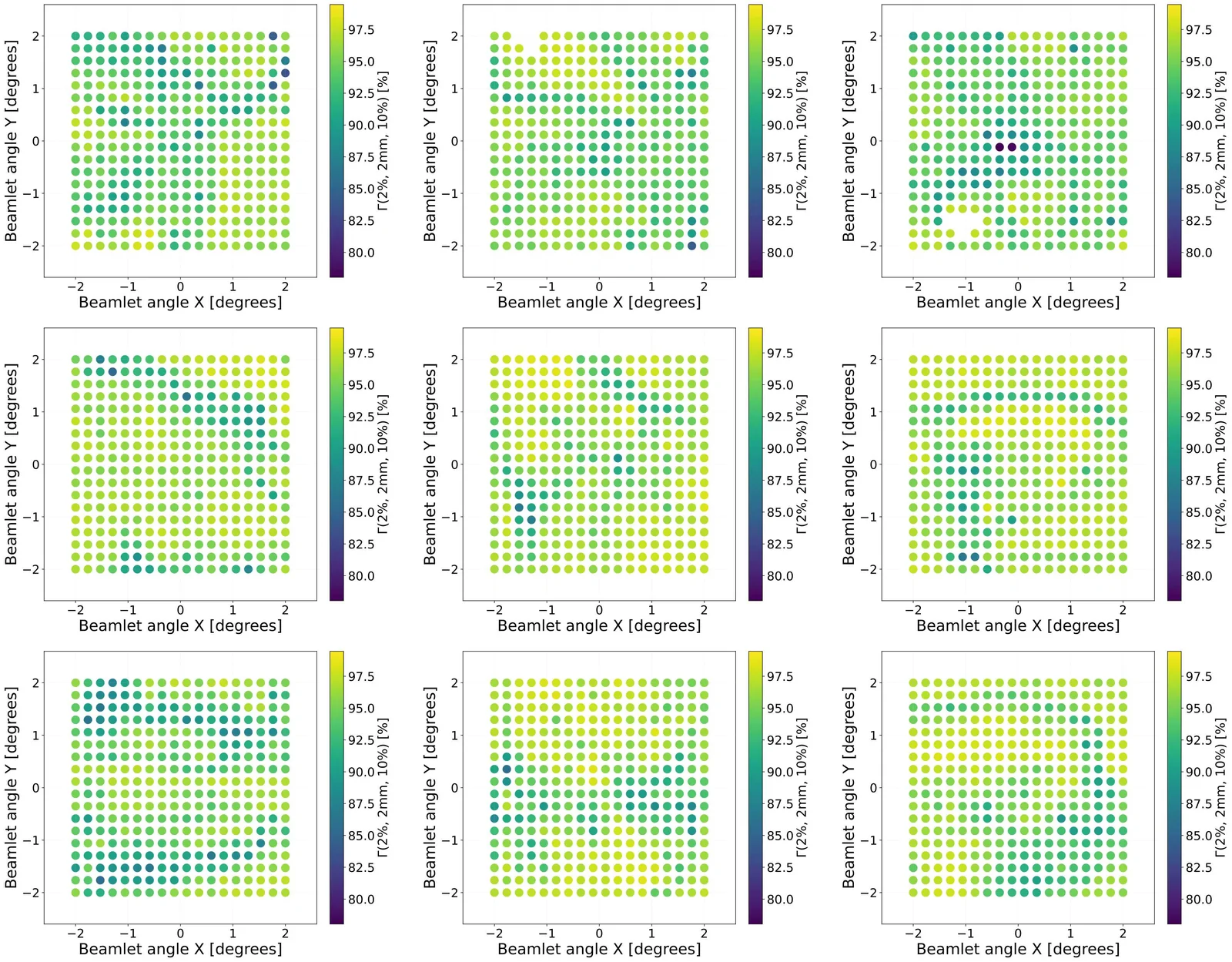
Per-patient, per-gantry Γ(2%, 2 mm, 10%) pass rates of the thoracic cohort at *ϵ* = 102.6 MeV, underlying the aggregate panel of Figure 4b. Layout as in Figure A3. Rows, top to bottom: patients Lung_Dx-G0037, G0049, and G0056; within each row, that patient’s 3 gantry angles in ascending order (G0037: 87.3^∘^, 137.9^∘^, 197.2^∘^; G0049: 183.2^∘^, 262.2^∘^, 317.5^∘^; G0056: 4.1^∘^, 80.6^∘^, 306.9^∘^). Missing cells correspond to beamlets excluded by the FD≥0.99 retention criterion.

**Figure A5:**
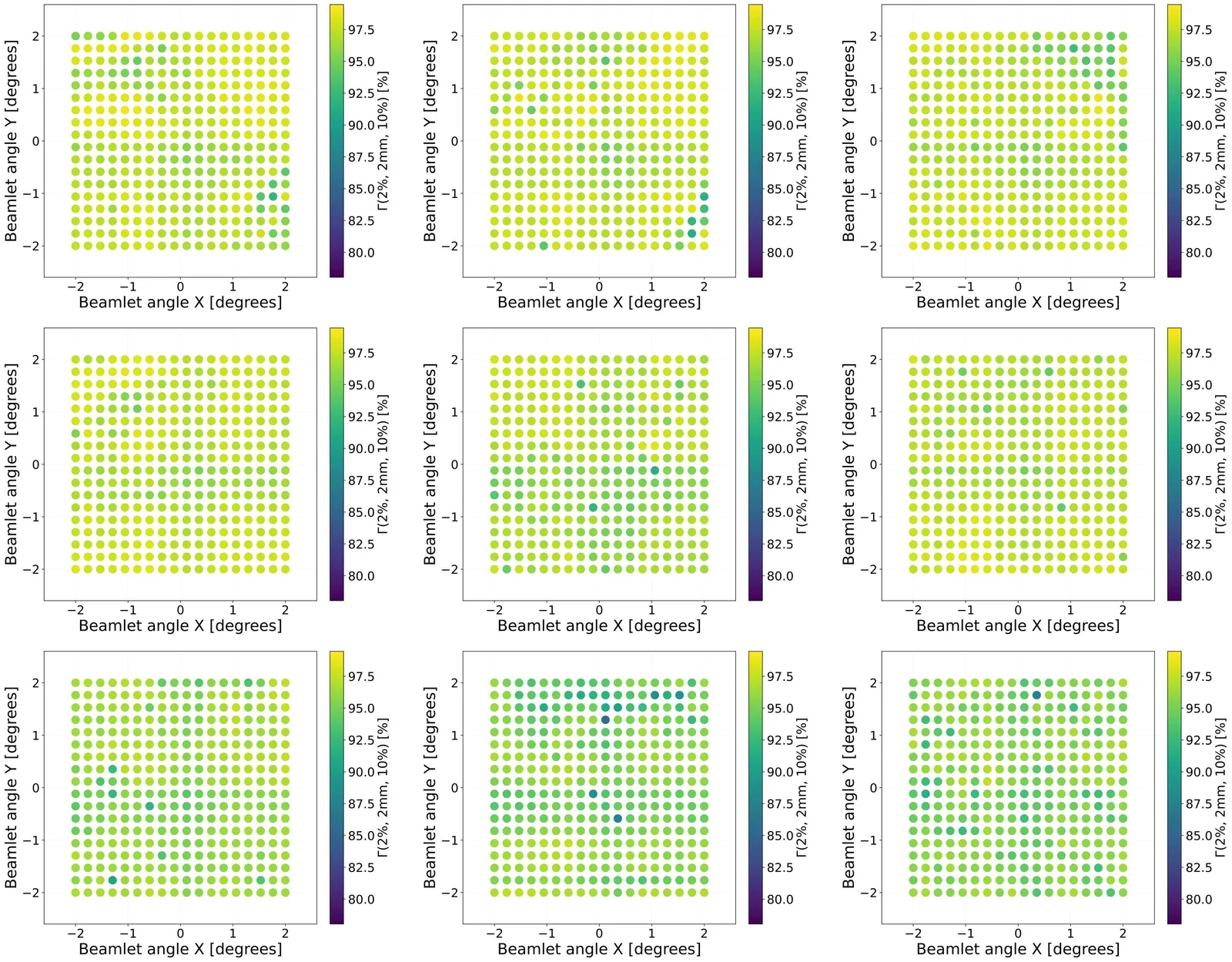
Per-patient, per-gantry Γ(2%, 2 mm, 10%) pass rates of the abdominal/pelvic cohort at *ϵ* = 135 MeV, underlying the aggregate panel of Figure 4c. Layout, patients, and gantry angles as in Figure A3.

**Figure A6:**
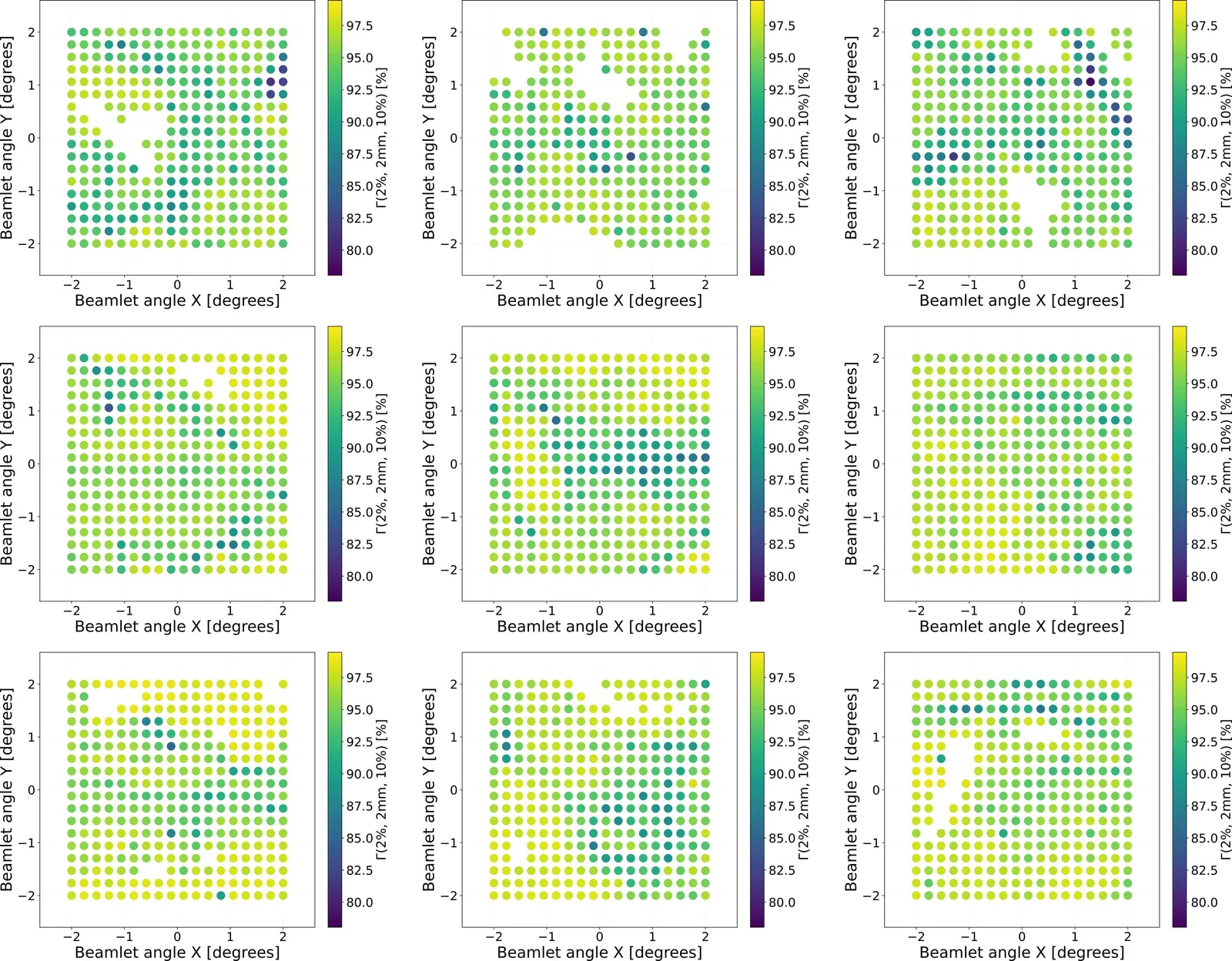
Per-patient, per-gantry Γ(2%, 2 mm, 10%) pass rates of the thoracic cohort at *ϵ* = 135 MeV, underlying the aggregate panel of Figure 4d. Layout, patients, and gantry angles as in Figure A4.

**Figure A7:**
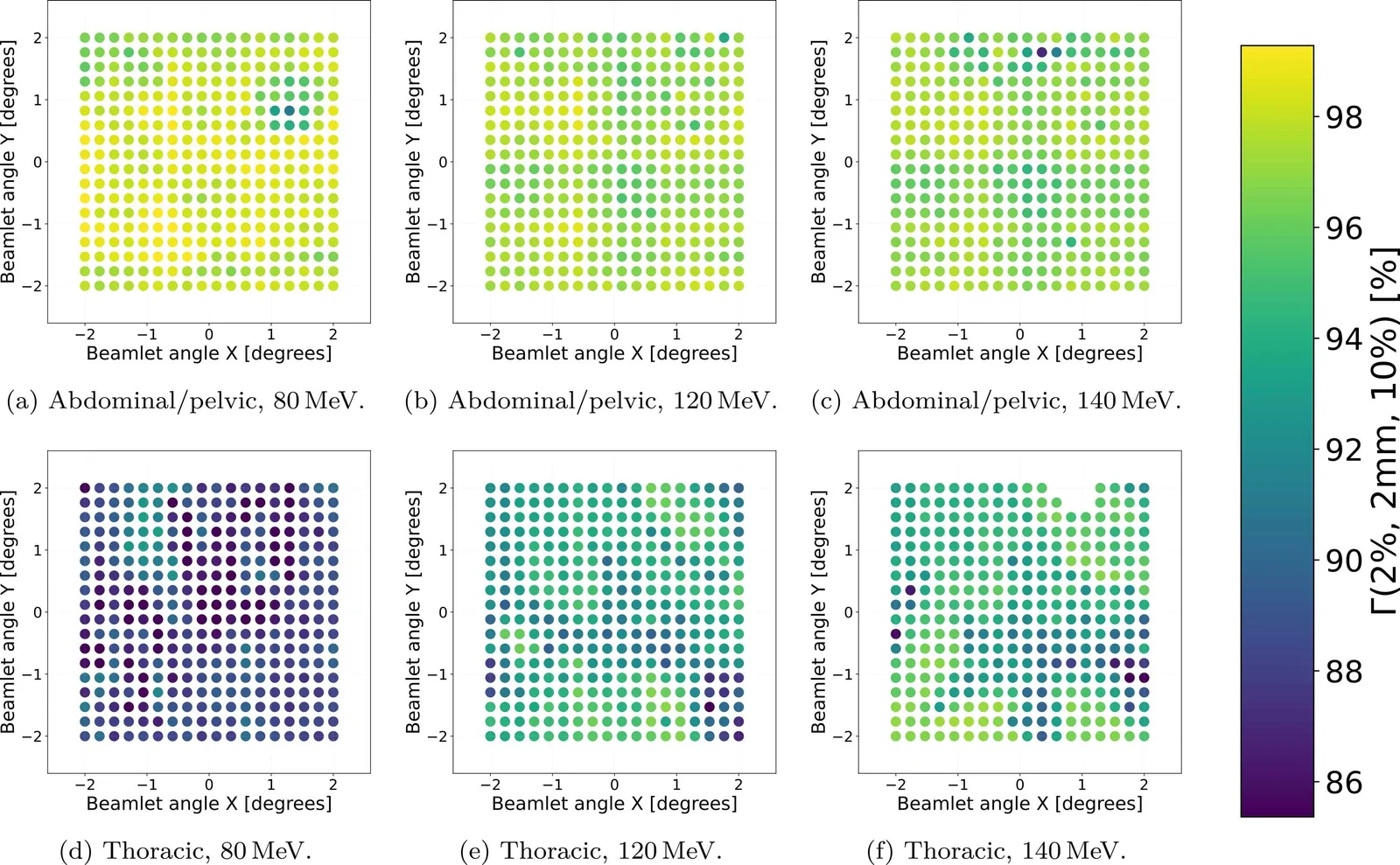
Extended angular robustness sweep under the Γ(2%, 2 mm, 10%) criterion on one additional abdominal/pelvic CT (top row) and one additional thoracic CT (bottom row) at 3 beam energies. Each panel shows the gamma pass rate for single beamlets scanned over lateral angles *θ*_*x*_ and *θ*_*y*_ in the range [−2^∘^,2^∘^] on an 18 × 18 grid, so each panel covers 324 scanned incidence angles. Beamlets whose fractional dose FD (Equation (3)) falls below 0.99 are omitted; this affects only panel f.

**Figure A8:**
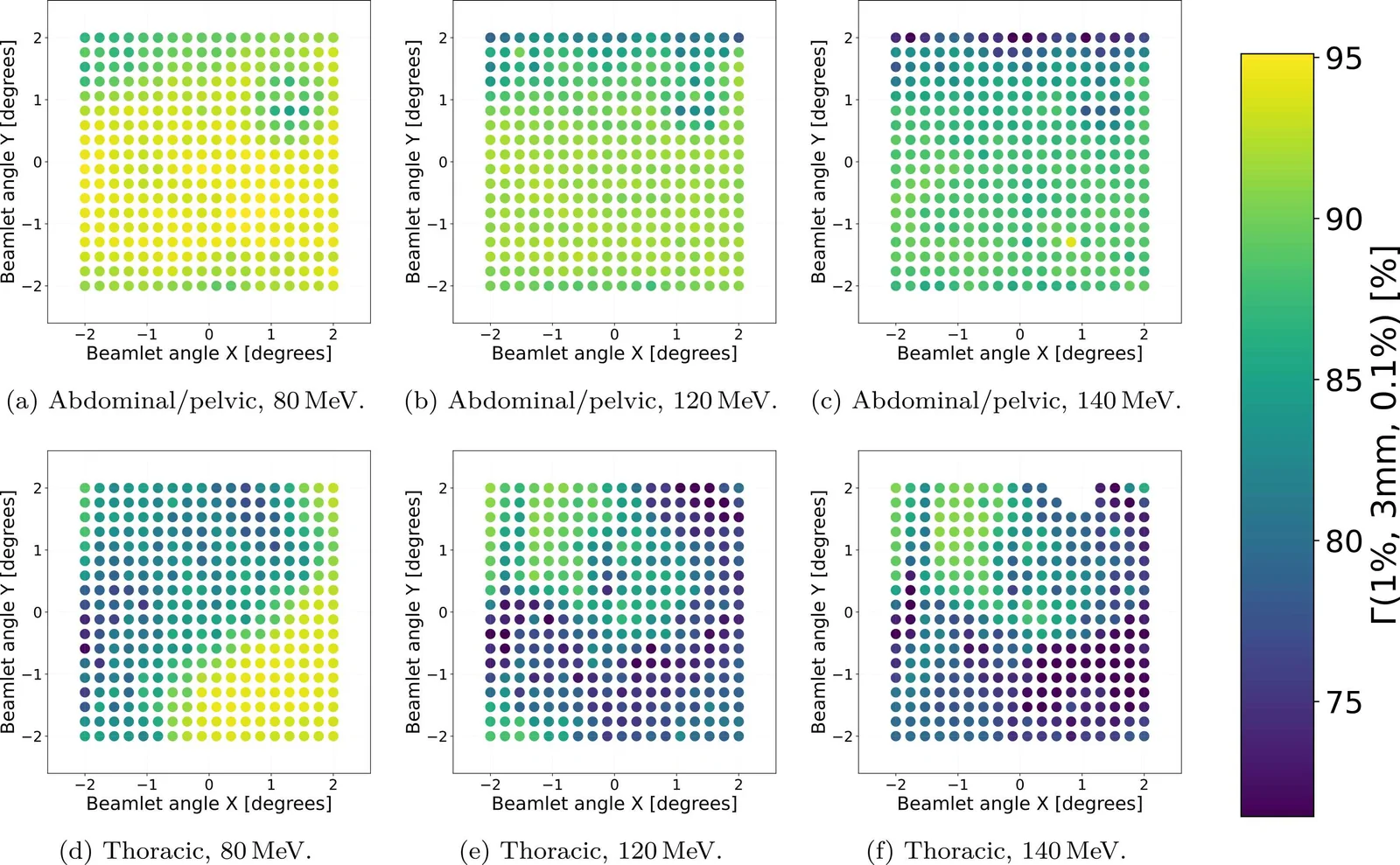
The same beamlets as in Figure A7, evaluated under the stricter Γ(1%, 3 mm, 0.1%) criterion, which applies a 0.1% rather than a 10% low-dose cutoff and therefore includes the low-dose halo surrounding each beamlet. As in Figure A7, beamlets failing the 0.99 dose-retention criterion of Equation (3) are omitted, which leaves the 140 MeV thoracic panel partly unpopulated. All 6 panels share the common color scale shown at the right.

**Figure A9:**
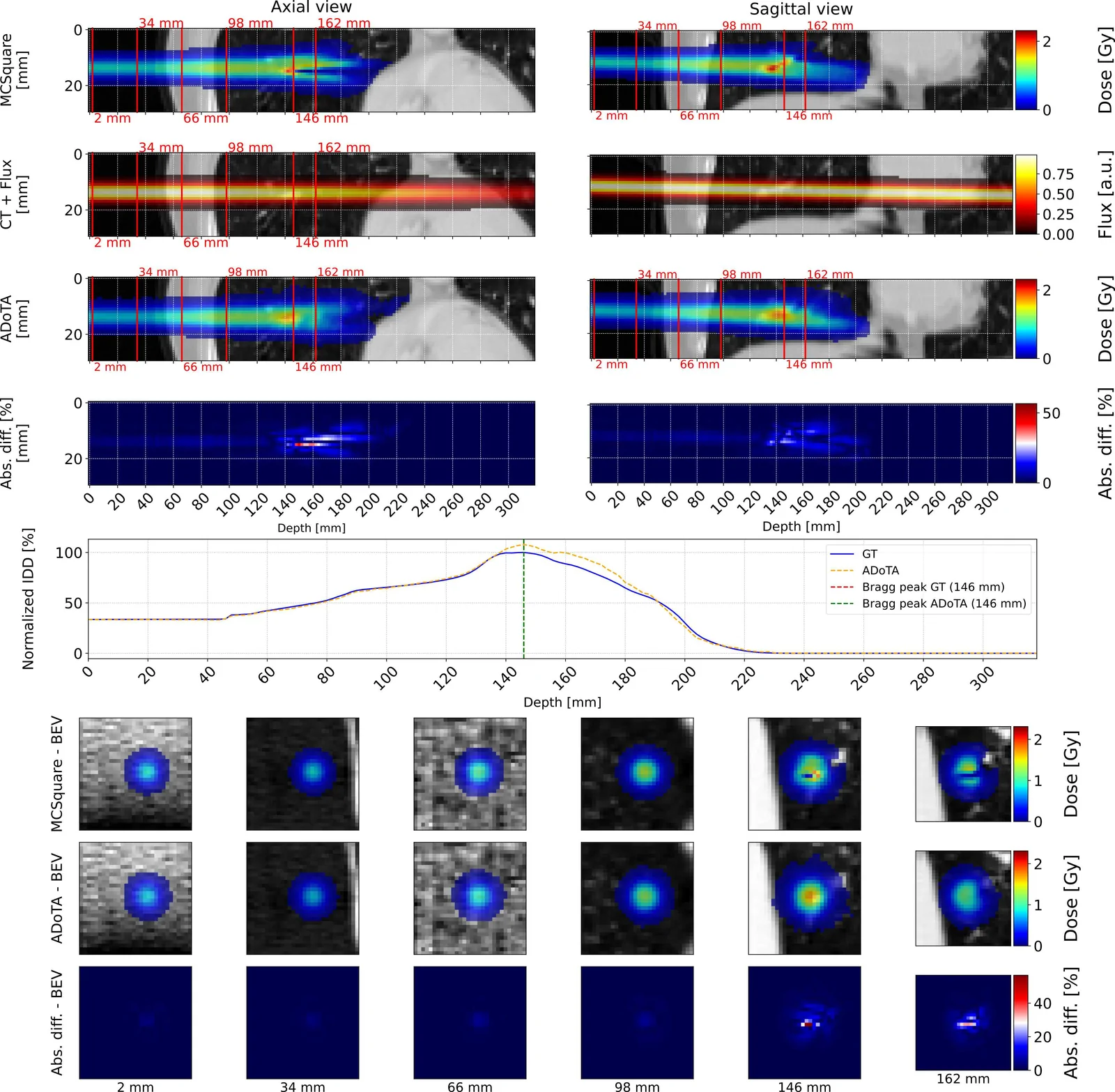
Worst-performing thoracic beamlet of the extended angular sweep, at 80 MeV, with Γ(2%, 2 mm, 10%) ≈ 78%. The panel layout follows Figure A15: the first 3 rows show the MCsquare reference dose, the ADoTA prediction, and their absolute percentage difference in axial and sagittal views; the fourth row shows the normalized integrated depth-dose profiles; and the final 3 rows show the beam’s eye view at the depths marked in red above. This layout is shared by Figure A9, Figure A10, Figure A11, Figure A12.

**Figure A10:**
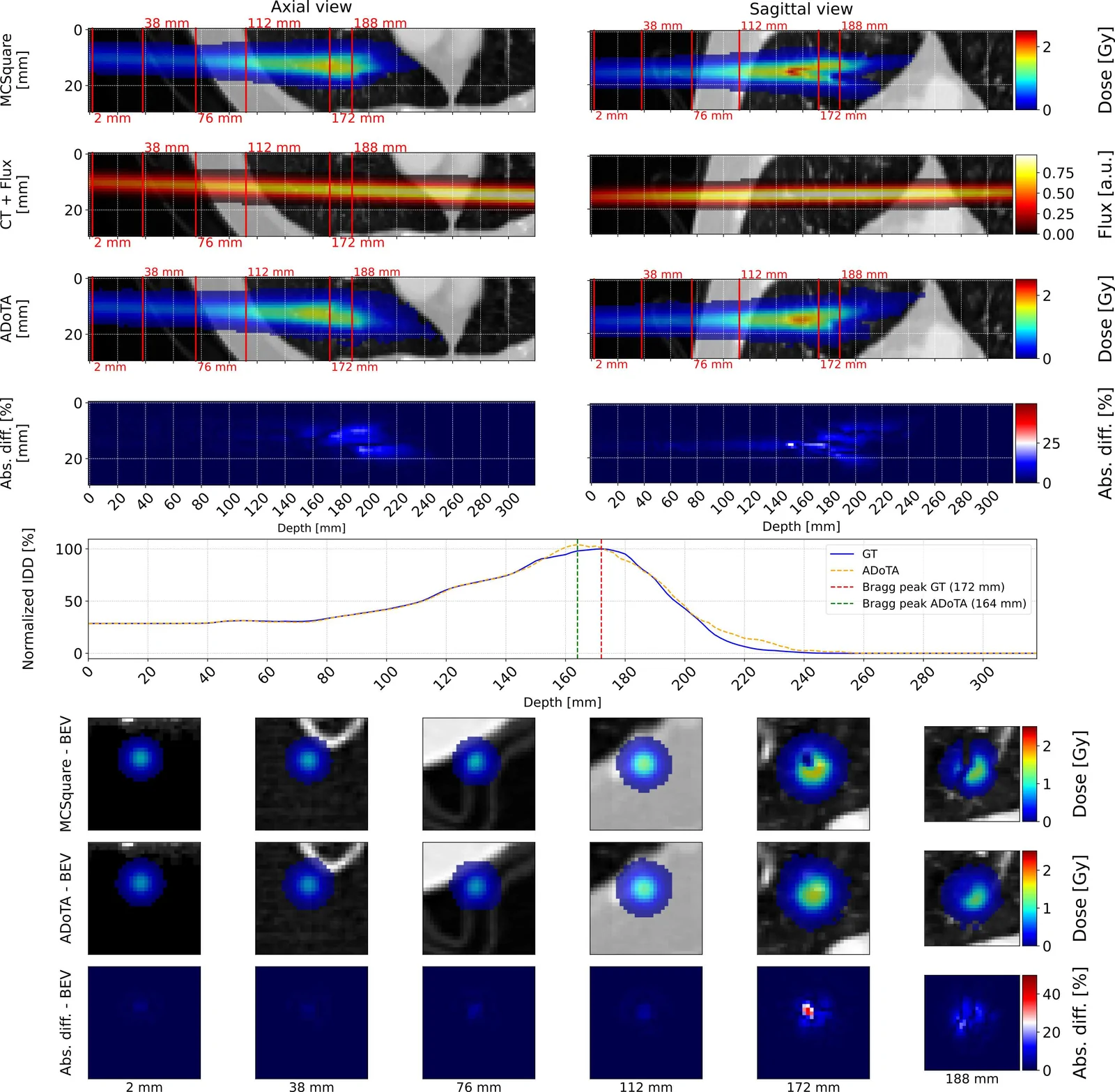
Second worst-performing thoracic beamlet of the extended angular sweep, at 80 MeV, with Γ(2%, 2 mm, 10%) ≈ 81%.

**Figure A11:**
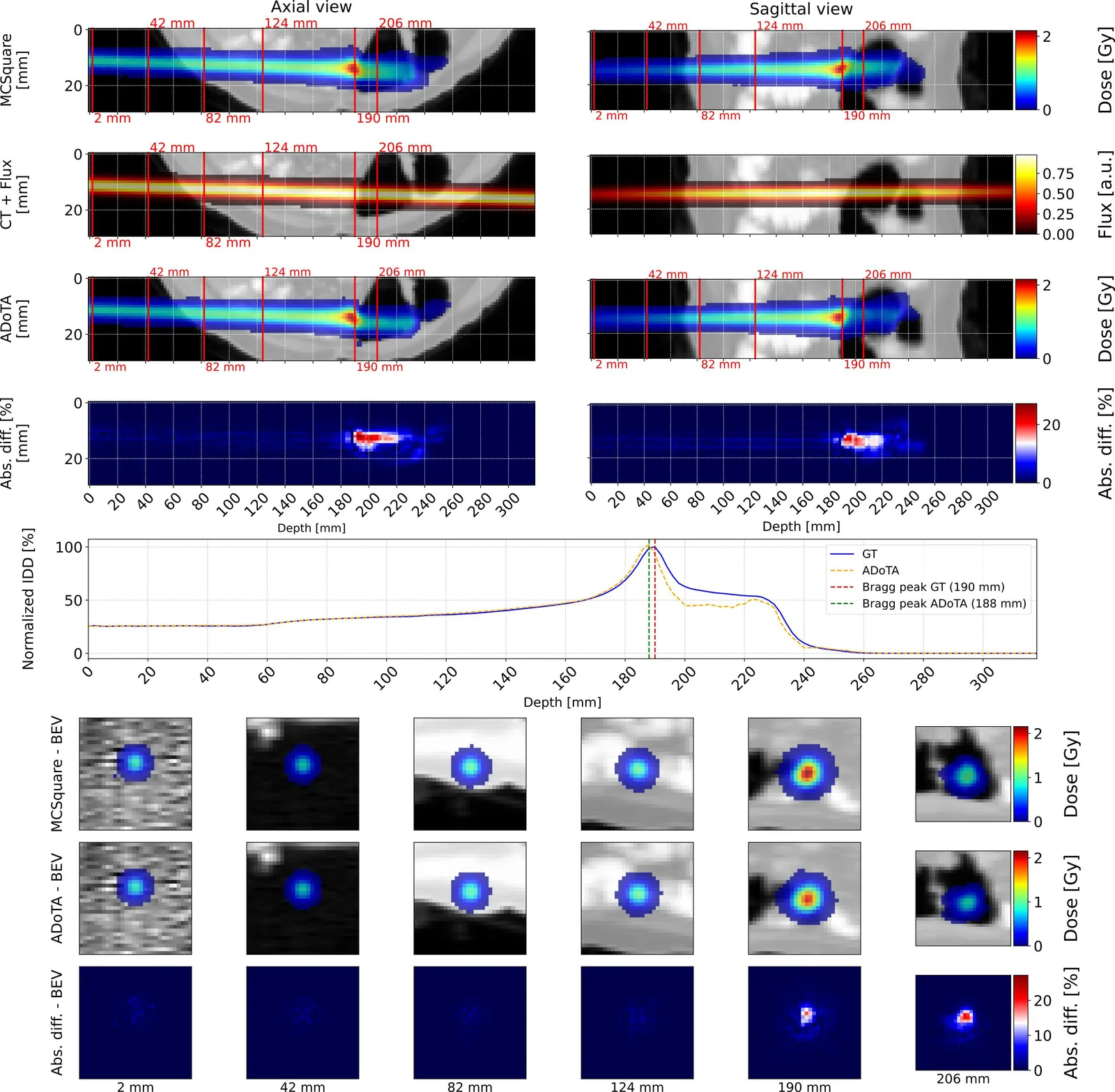
Worst-performing abdominal/pelvic beamlet of the extended angular sweep, at 140 MeV, with Γ(2%, 2 mm, 10%) ≈ 86%.

**Figure A12:**
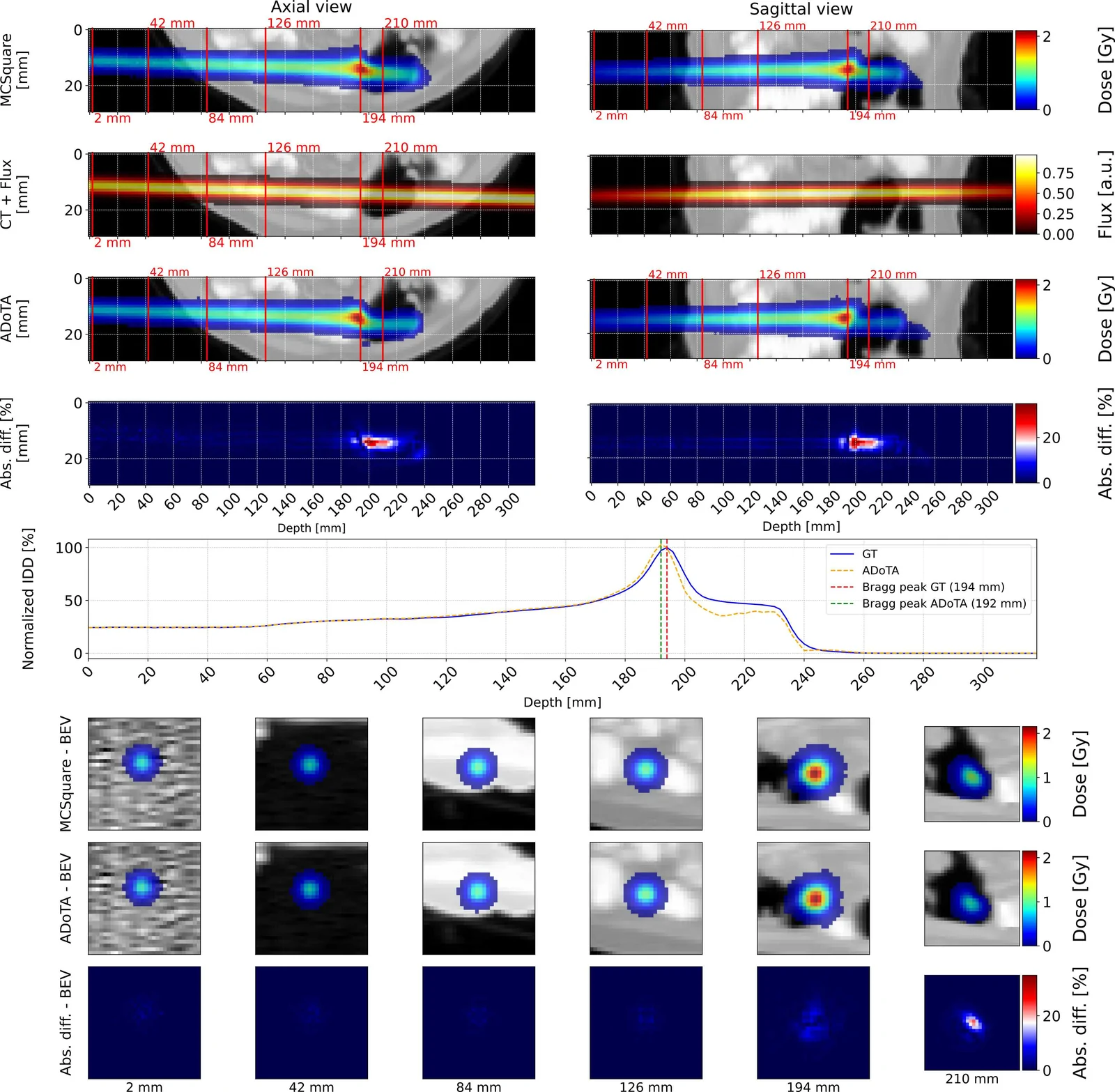
Second worst-performing abdominal/pelvic beamlet of the extended angular sweep, at 140 MeV, with Γ(2%, 2 mm, 10%) ≈ 91%. A second beamlet, at 80 MeV, attains the same pass rate to the reported precision.

**Figure A13:**
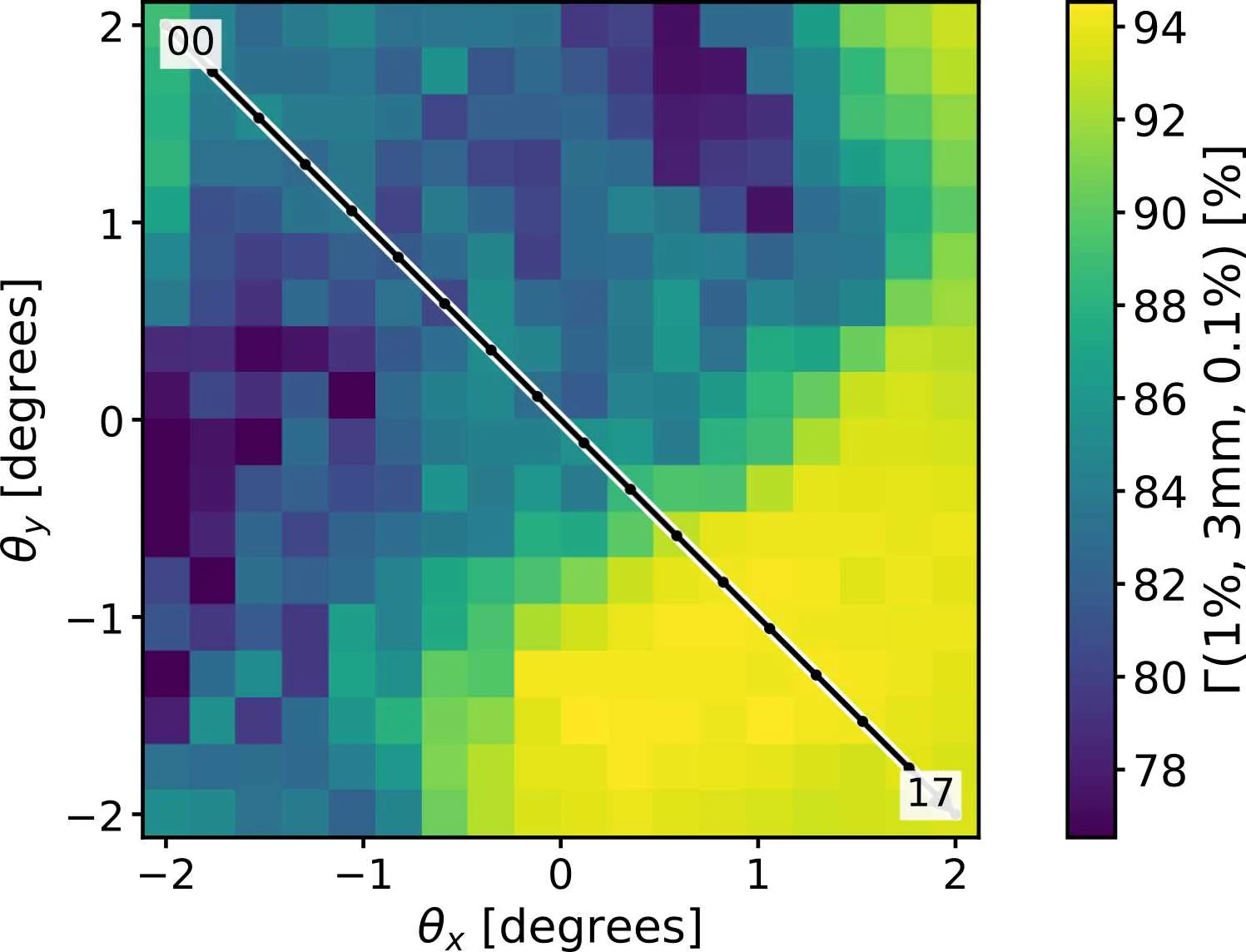
Sampling of the anti-diagonal of the thoracic 80 MeV angular grid. The map reproduces the corresponding panel of Figure A8 under the Γ(1%, 3 mm, 0.1%) criterion. The 18 sampled positions run from 00 at (*θ*_*x*_, *θ*_*y*_) = −2^∘^, +2^∘^) to 17 at +2^∘^, −2^∘^); each is one beamlet of the existing sweep.

**Figure A14:**
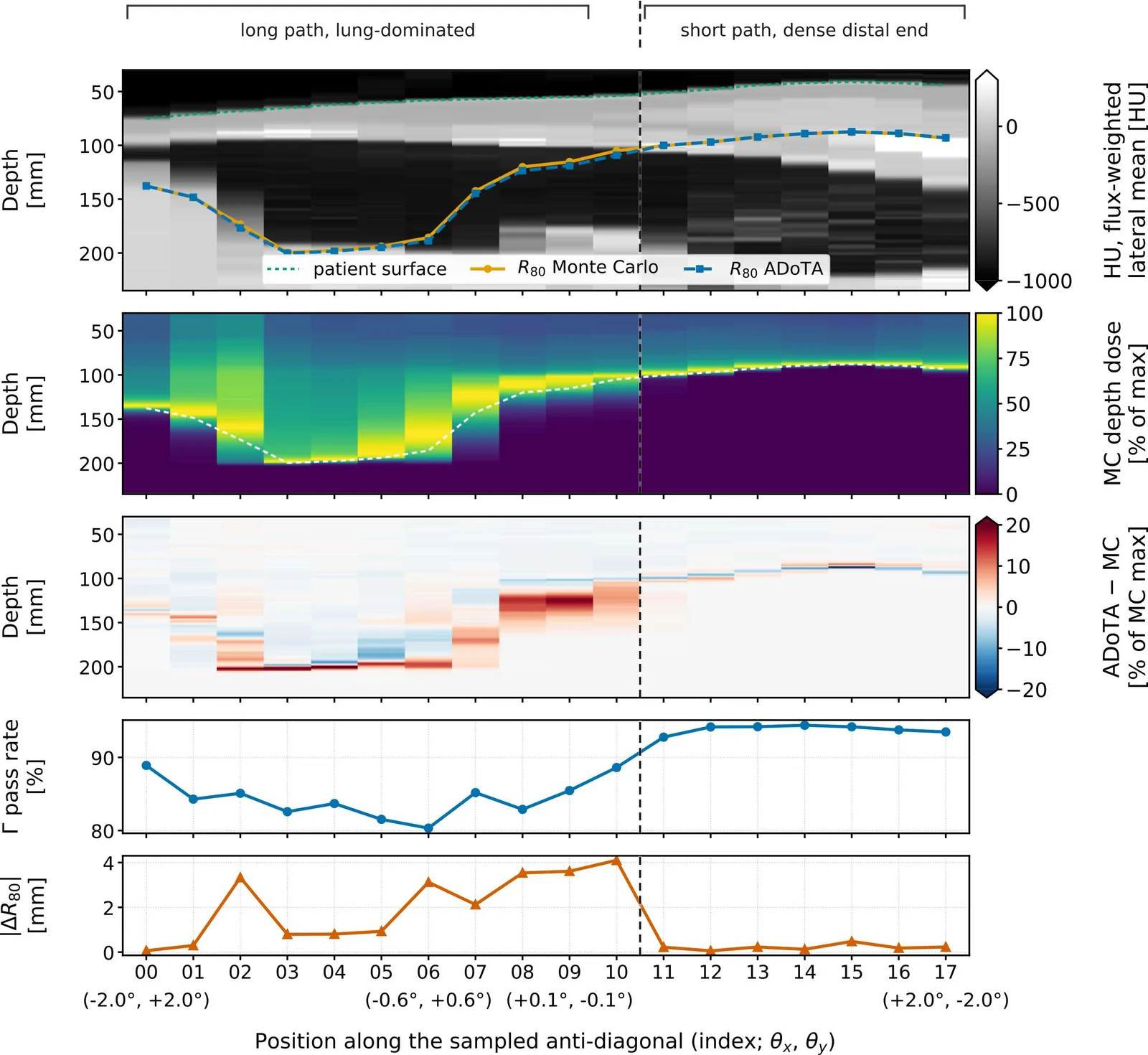
Anatomy, dose, and prediction error along the anti-diagonal sampled in Figure A13, against position on that diagonal. From top to bottom: the flux-weighted lateral mean CT number at each depth, with the patient surface and the Monte Carlo and ADoTA *R*_80_ depths; the laterally integrated Monte Carlo depth dose, normalized to its own maximum; the same integrated profile for ADoTA minus Monte Carlo, normalized to that same maximum so both dose panels share one scale per position; the gamma pass rate Γ(1%, 3 mm, 0.1%), evaluated on the full 3-dimensional dose rather than on the integrated profile; and the absolute distal range error ∣Δ*R*_80_∣. The dashed line marks the transition at position 10/11 between the long, lung-dominated paths and the shorter paths ending in dense tissue.

**Figure A15:**
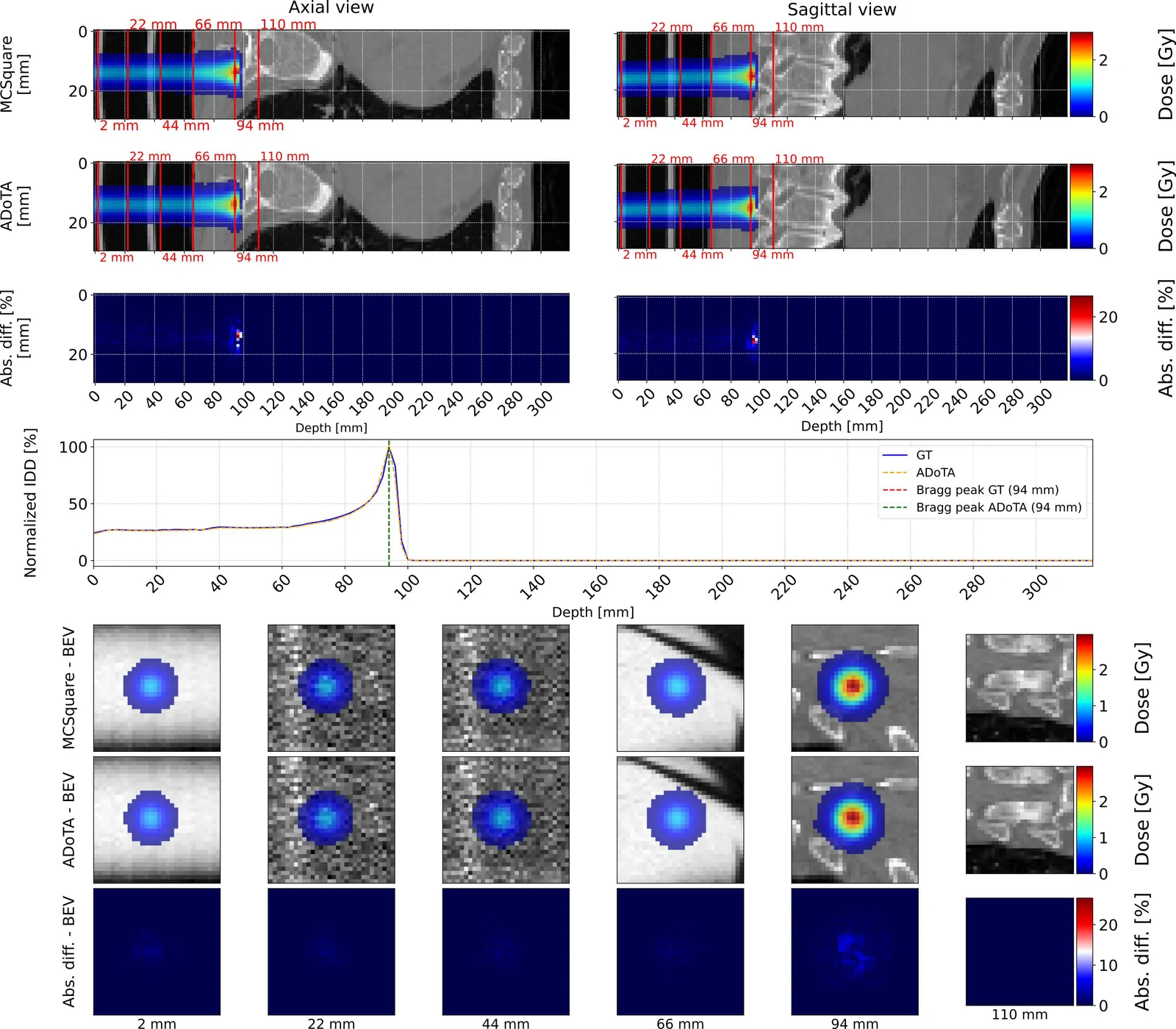
The best performing case in the lung test set. Beamlet initial energy: 76.68 MeV. MAPE = 3.84%, Γ(2%, 2 mm, 10%) = 100.0%. The first 3 rows display the 3D dose distributions calculated by MCsquare (first row) and ADoTA (second row), along with the absolute percentage difference between them (third row). The axial and sagittal planes are centered on the voxel containing the Bragg peak. The fourth row presents the normalized IDD profiles for both the MCsquare and ADoTA distributions. The final 3 rows show the beam’s eye view at selected depths, as indicated by the red markers in the upper rows. Only voxels receiving a dose above 10 % of the maximum deposited dose are included in the MAPE calculation. This visualization layout is consistent for Figures A15-A18.

**Figure A16:**
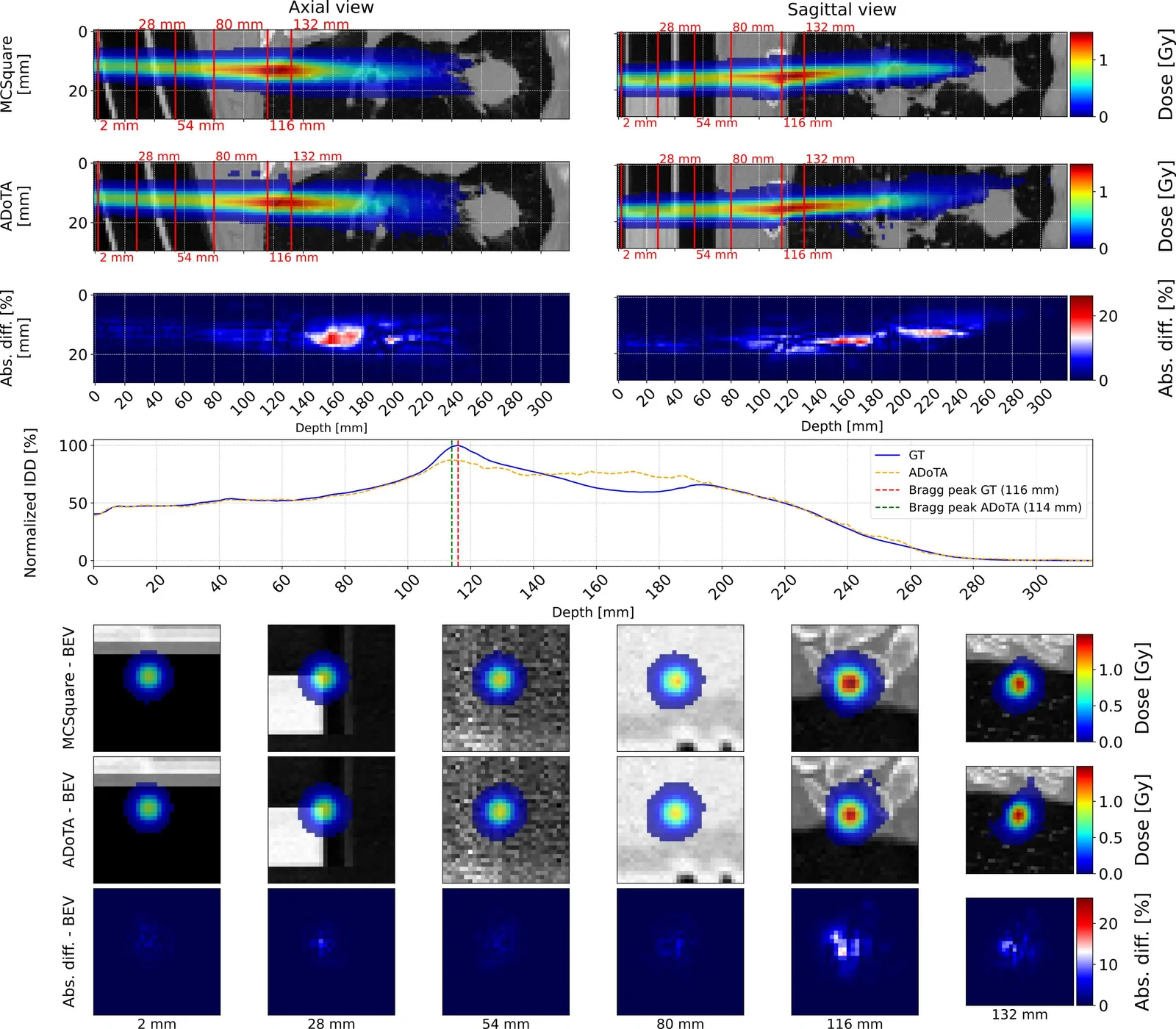
The worst-performing case in the lung test set. Beamlet initial energy: 111.23 MeV. MAPE = 12.99%, Γ(2%, 2 mm, 10%) = 89.61%.

**Figure A17:**
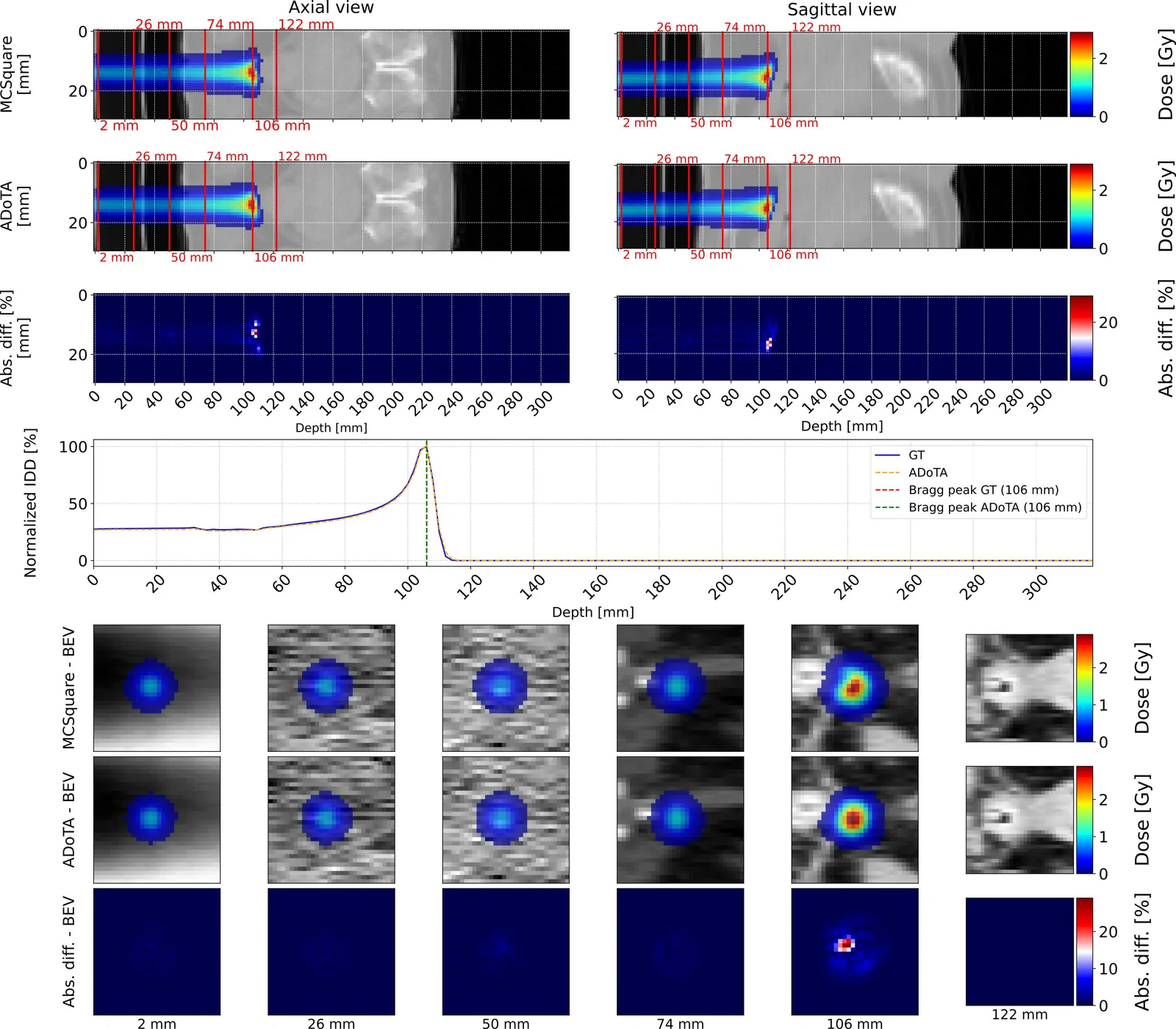
The best-performing case from the abdominal/pelvic test set. Beamlet initial energy: 86.71 MeV. MAPE = 4.63%, Γ(2%, 2 mm, 10%) = 100.0%.

**Figure A18:**
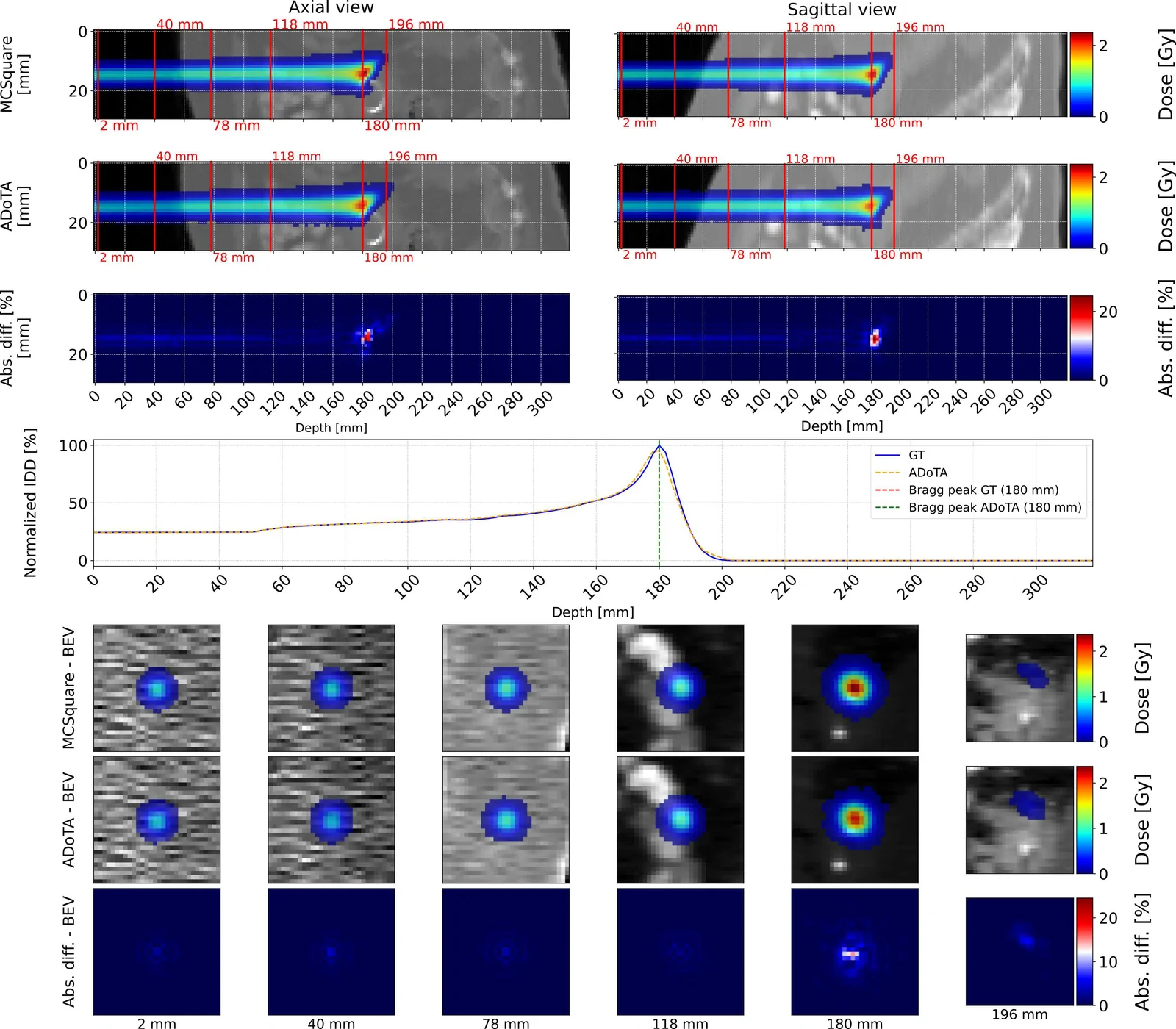
The worst-performing case from the abdominal/pelvic test set. Beamlet initial energy: 136.68 MeV; MAPE = 4.44%, Γ(2%, 2 mm, 10%) = 95.64%.

**Figure A19:**
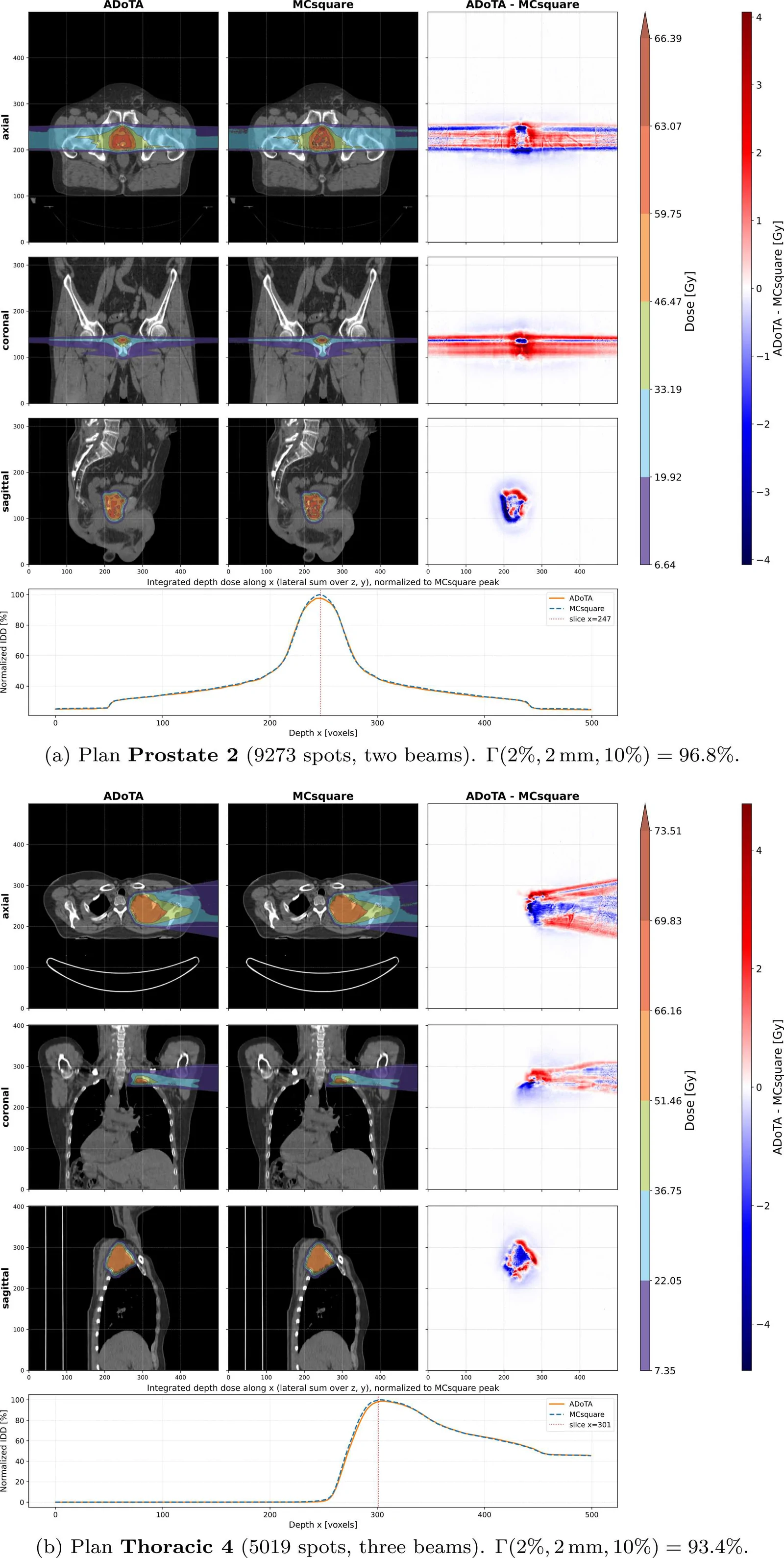
Qualitative full-plan dose comparison between ADoTA and the MCsquare reference for 2 representative plans, selected from the 8-plan test set of Table 2a. For each plan, the panels show axial, coronal, and sagittal slices (rows) of the MCsquare reference dose, the ADoTA prediction, and their voxel-wise difference (columns), with the laterally integrated depth-dose profile beneath. The corresponding dose-volume histograms are shown in Figures A20 and A21.

**Figure A20:**
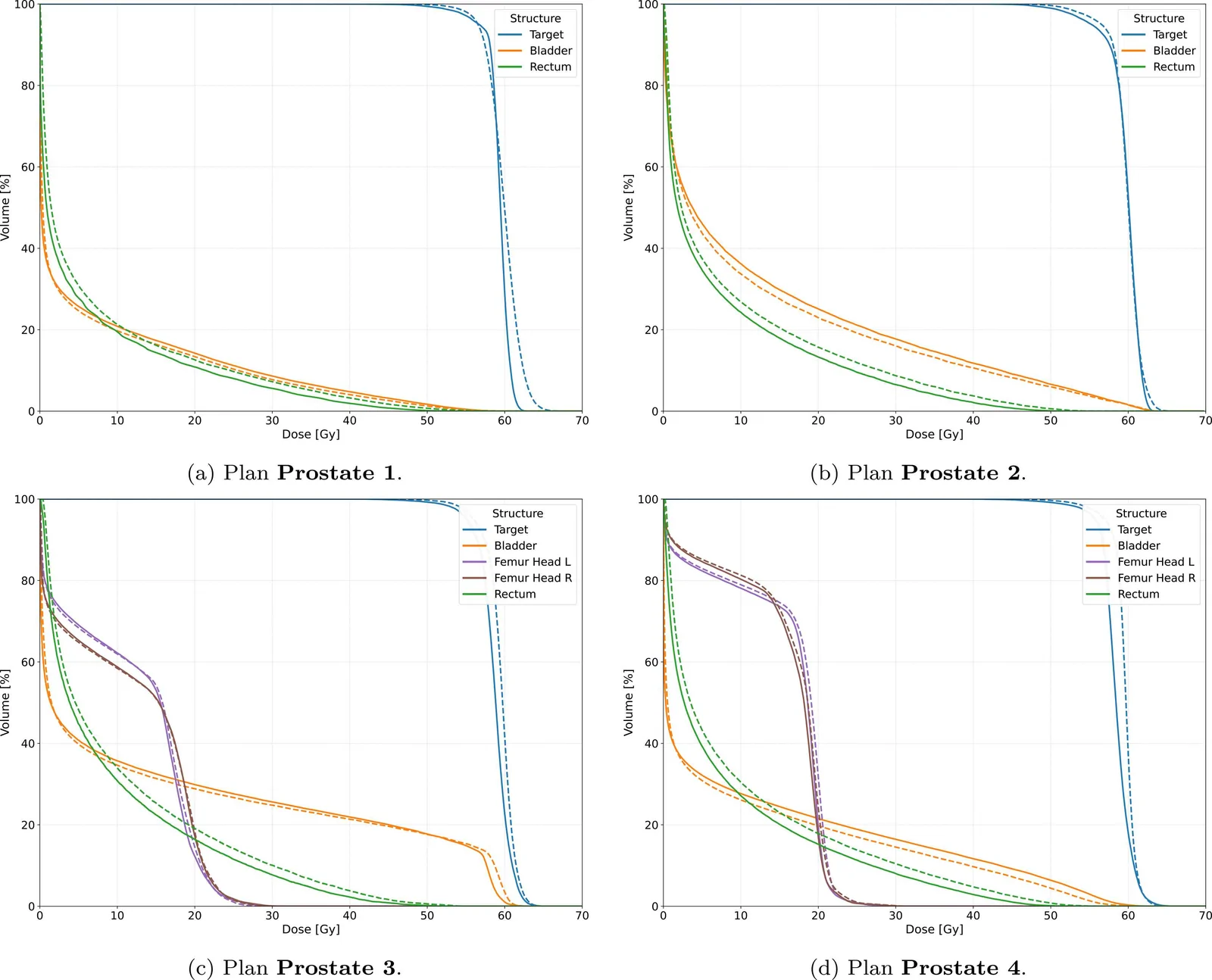
Dose-volume histograms for the 4 prostate plans in Table 2a, organs at risk labeled by anatomical name. Each panel compares ADoTA (solid) with MCsquare (dashed) for the target and organs at risk.

**Figure A21:**
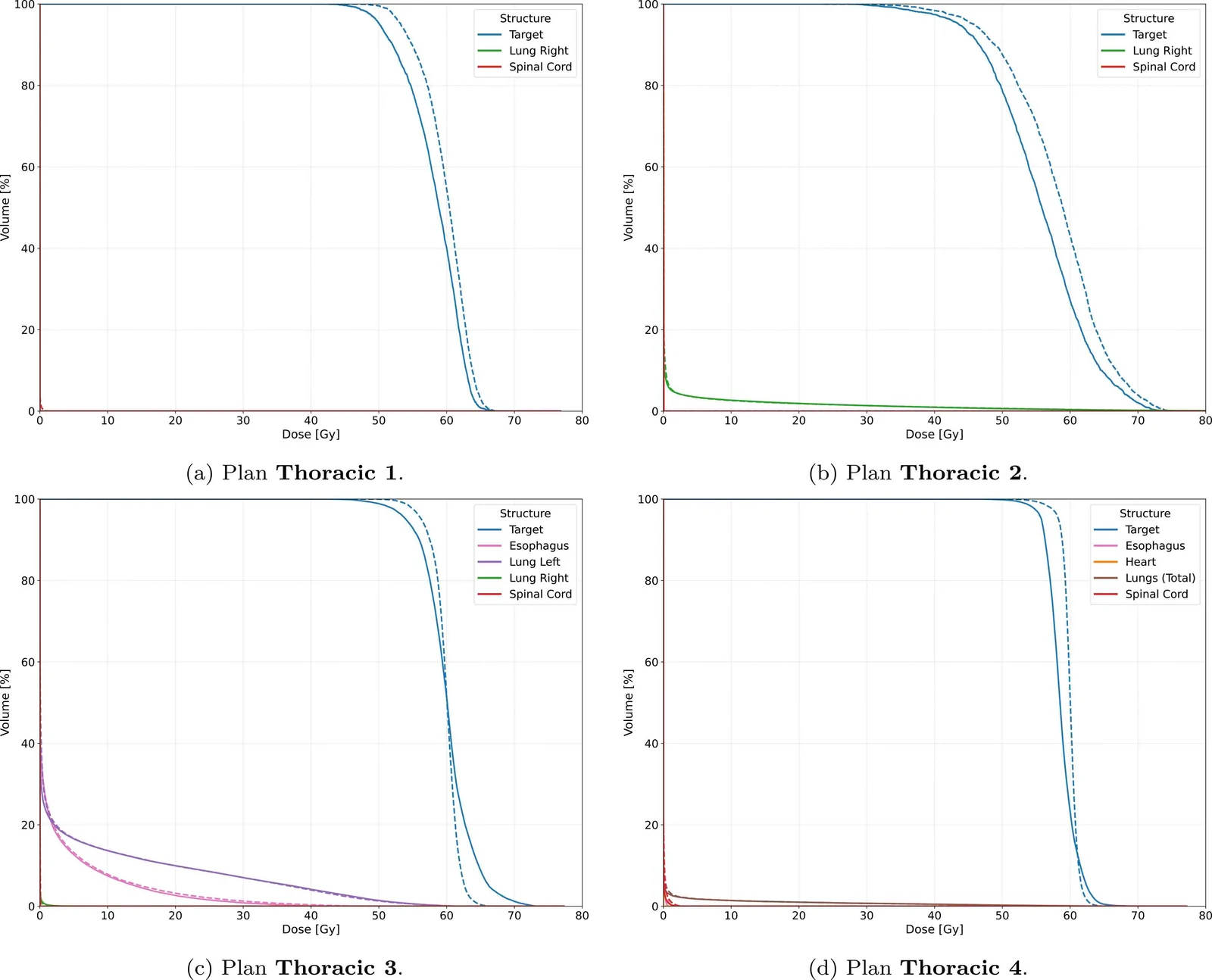
Dose-volume histograms for the 4 thoracic plans in Table 2a, organs at risk labeled by anatomical name. Each panel compares ADoTA (solid) with MCsquare (dashed) for the target and organs at risk.

**Table A4:** Combined inference-time comparison across dose calculation models and ADoTA batch-scaling analysis.

Model	System	*B*	*T*_batch_ (ms)	Beamlet mean (ms)	Std (ms)	Fastest (ms)	Slowest (ms)
				*Cross-model comparison (effective beamlet inference time)*			
ADoTA (this work)	b	56	187.1	3.34	0.08	3.3	3.64
CC-LSTM Neishabouri et al^30^	a	–	–	3.6	–	–	–
DoTA Pastor-Serrano and Perko^8^	b	–	–	5.0	4.9	–	–
MED-LSTM Pang et al^29^	a	1	200.0	200.0	–	–	–
PBA Cisternas et al^50^	c	–	–	728.3	30.9	–	–
MC Souris et al^36^	c	–	–	43 636.9	12 291.6	–	–
				*ADoTA batch-scaling on individualized beamlet-level test set (this work)*			
ADoTA (this work)	b	1	14.1	14.1	10.2	13.5	326.6
		8	35.9	4.48	0.275	4.4	7.2
		16	60.2	3.76	0.325	1.57	5.01
		32	110.4	3.45	0.15	2.85	4.06
		56	187.1	3.34	0.08	3.3	3.64
		200	628.1	3.14	0.31	2.51	3.36

Reported times exclude preprocessing and postprocessing. For ADoTA, *batch inference time* is the forward-pass latency for batch size *B*, and *beamlet inference time* is *T*_batch_∕*B*, that is, an effective throughput rather than the latency of an isolated prediction; the latter is given by the *B* = 1 row (14.1 ms). Timing dispersion (Std/Fastest/Slowest) is reported for every ADoTA batch size. The published values in the upper block were obtained on different hardware, with different batch sizes, numerical precision, and timing definitions, and are reproduced here as indicative context rather than as a controlled comparison; no ranking between models should be inferred from them.

a Nvidia Quadro RTX 6000, 64 GB RAM.

b 4 vCPUs, Nvidia A40, 40 GB RAM.

c 8 CPUs, Intel Xeon E5–2620, 16 GB RAM.

**Table A5:** Per-beamlet direction-encoding cost across the GPU-batched sweep, measured on a single NVIDIA A40 GPU using single precision (32 bit floating point).

		Compute only [ms]			Including host-device transfer [ms]	
Batch size	ADoTA	DoTA-like	Ratio	ADoTA	DoTA-like	Ratio
1	1.671	1.447	0.9	1.814	3.199	1.8
8	0.237	0.294	1.2	0.352	1.433	4.1
16	0.146	0.250	1.7	0.247	1.260	5.1
32	0.107	0.334	3.1	0.208	1.270	6.1
56	0.085	0.339	4.0	0.177	1.232	6.9
112	0.076	0.346	4.5	0.475	1.841	3.9

Each entry is the mean cost per beamlet in milliseconds; the ratio is the DoTA-like cost divided by the ADoTA cost, so values above one favor the analytical projection. The batch-independent single-threaded CPU baseline is 9.06 ms/beamlet for the analytical projection and 48.10 ms/beamlet for the 2 resampling operations.

**Table A6:** Measured plan compute time (excludes disk I/O and data loading) for the 2 routes on the full benchmark plan (5640 spots, 2 fields) by pipeline stage.

Stage	ADoTA [s]	DoTA-like [s]
**Direction encoding**	**2.64**	25.46
	*Stages common to both routes*	
Field rotation and de-rotation	0.44	0.39
Crop	4.51	3.91
Prep	5.63	4.02
Forward (model)	28.19	28.18
Post-processing	0.54	0.51
Dose deposit	1.11	1.11
Subtotal	40.43	38.11
Total (compute-only)	43.07	63.57

Direction encoding, the only stage whose function differs between the routes, is the analytical projection for ADoTA and the 2 beamlet resampling passes for the DoTA-like route. The remaining stages perform the same work in both routes and are listed below it.

**Table A7:** Detailed per-plan dose-volume metrics, extending the summary in Table 2a.

Plan Identifier			Prostate				Thoracic	
	1	2	3	4	1	2	3	4
*D*_98%_ (target), MC / ADoTA	55.1 / 54.0	53.5 / 51.9	54.3 / 53.2	54.7 / 53.5	51.8 / 48.3	41.4 / 38.1	54.7 / 51.6	57.0 / 54.4
Δ*D*_98%_ (target) [%]	−1.8	−2.5	−1.9	−2.0	−5.9	−5.4	−5.2	−4.2
*D*_95%_ (target), MC / ADoTA	56.5 / 56.7	56.5 / 55.2	56.0 / 55.1	57.0 / 55.8	53.2 / 50.1	45.8 / 43.5	56.3 / 54.0	58.3 / 55.8
Δ*D*_95%_ (target) [%]	+0.2	−2.1	−1.5	−2.0	−5.2	−3.8	−3.8	−4.2
*D*_2%_ (target), MC / ADoTA	64.0 / 61.5	63.2 / 62.4	62.7 / 62.3	62.3 / 62.3	65.3 / 64.3	71.8 / 70.1	63.5 / 68.8	62.3 / 63.5
Δ*D*_2%_ (target) [%]	−4.0	−1.2	−0.6	+0.1	−1.7	−2.8	+8.8	+2.0
*D*_mean_ (Bladder), MC / ADoTA	6.3 / 6.7	12.1 / 12.9	15.8 / 16.0	9.4 / 10.4	–	–	–	–
Δ*D*_mean_ (Bladder) [%]	+0.6	+1.4	+0.2	+1.5	–	–	–	–
*D*_mean_ (Rectum), MC / ADoTA	7.0 / 5.9	8.4 / 7.3	10.3 / 9.1	9.7 / 8.3	–	–	–	–
Δ*D*_mean_ (Rectum) [%]	−1.8	−2.0	−2.1	−2.4	–	–	–	–
*D*_mean_ (Femur Head R), MC / ADoTA	–	–	11.6 / 11.6	15.7 / 15.2	–	–	–	–
Δ*D*_mean_ (Femur Head R) [%]	–	–	+0.0	−0.7	–	–	–	–
*D*_mean_ (Femur Head L), MC / ADoTA	–	–	11.9 / 11.8	15.7 / 15.2	–	–	–	–
Δ*D*_mean_ (Femur Head L) [%]	–	–	−0.2	−0.8	–	–	–	–
*D*_mean_ (Spinal Cord), MC / ADoTA	–	–	–	–	0.0 / 0.0	0.0 / 0.0	0.0 / 0.0	0.1 / 0.0
Δ*D*_mean_ (Spinal Cord) [%]	–	–	–	–	−0.0	+0.0	−0.0	−0.1
*D*_mean_ (Lung Right), MC / ADoTA	–	–	–	–	0.0 / 0.0	1.1 / 1.1	0.0 / 0.0	–
Δ*D*_mean_ (Lung Right) [%]	–	–	–	–	+0.0	−0.0	+0.0	–
*D*_mean_ (Lung Left), MC / ADoTA	–	–	–	–	–	–	4.7 / 4.7	–
Δ*D*_mean_ (Lung Left) [%]	–	–	–	–	–	–	−0.1	–
*D*_mean_ (Lungs (Total)), MC / ADoTA	–	–	–	–	–	–	–	0.5 / 0.5
Δ*D*_mean_ (Lungs (Total)) [%]	–	–	–	–	–	–	–	−0.0
*D*_mean_ (Esophagus), MC / ADoTA	–	–	–	–	–	–	2.3 / 2.2	0.1 / 0.0
Δ*D*_mean_ (Esophagus) [%]	–	–	–	–	–	–	−0.3	−0.1
*D*_mean_ (Heart), MC / ADoTA	–	–	–	–	–	–	–	0.0 / 0.0
Δ*D*_mean_ (Heart) [%]	–	–	–	–	–	–	–	−0.0

Absolute MC / ADoTA values are in Gy(RBE); Δ rows are signed (ADoTA − MC) as a percentage of the prescribed dose. A dash indicates the structure is not present in that plan.

**Table A8:** Effect of FP16 versus FP32 inference precision on plan-level dose accuracy, for one representative prostate plan (Prostate 1) and one representative thoracic plan (Thoracic 1); all other pipeline settings held fixed.

		Prostate 1			Thoracic 1	
Metric	FP32	FP16	Δ	FP32	FP16	Δ
Γ(2%, 2 mm, 10%) [%]	94.757	94.762	+0.005	97.689	97.664	−0.025
Γ(3%, 3 mm, 10%) [%]	98.478	98.482	+0.004	99.553	99.540	−0.013
Γ(1%, 3 mm, 0.1%) [%]	95.708	95.706	−0.002	99.327	99.321	−0.006
RMSE [Gy]	1.8171	1.8167	−0.0004	0.5847	0.5856	+0.0009
*ρ* [%]	0.0379	0.0379	−0.0000	0.0089	0.0089	+0.0000
Network inference [s]	12.55	8.31	−4.24	1.12	0.95	−0.17
Streaming runtime [s]	33.22	28.22	−5.00	4.89	4.64	−0.25

Δ is FP16−FP32. Network inference and total streaming runtime are included for context, since they are the reason FP16 is used; neither is a dosimetric metric.

**Figure A22:**
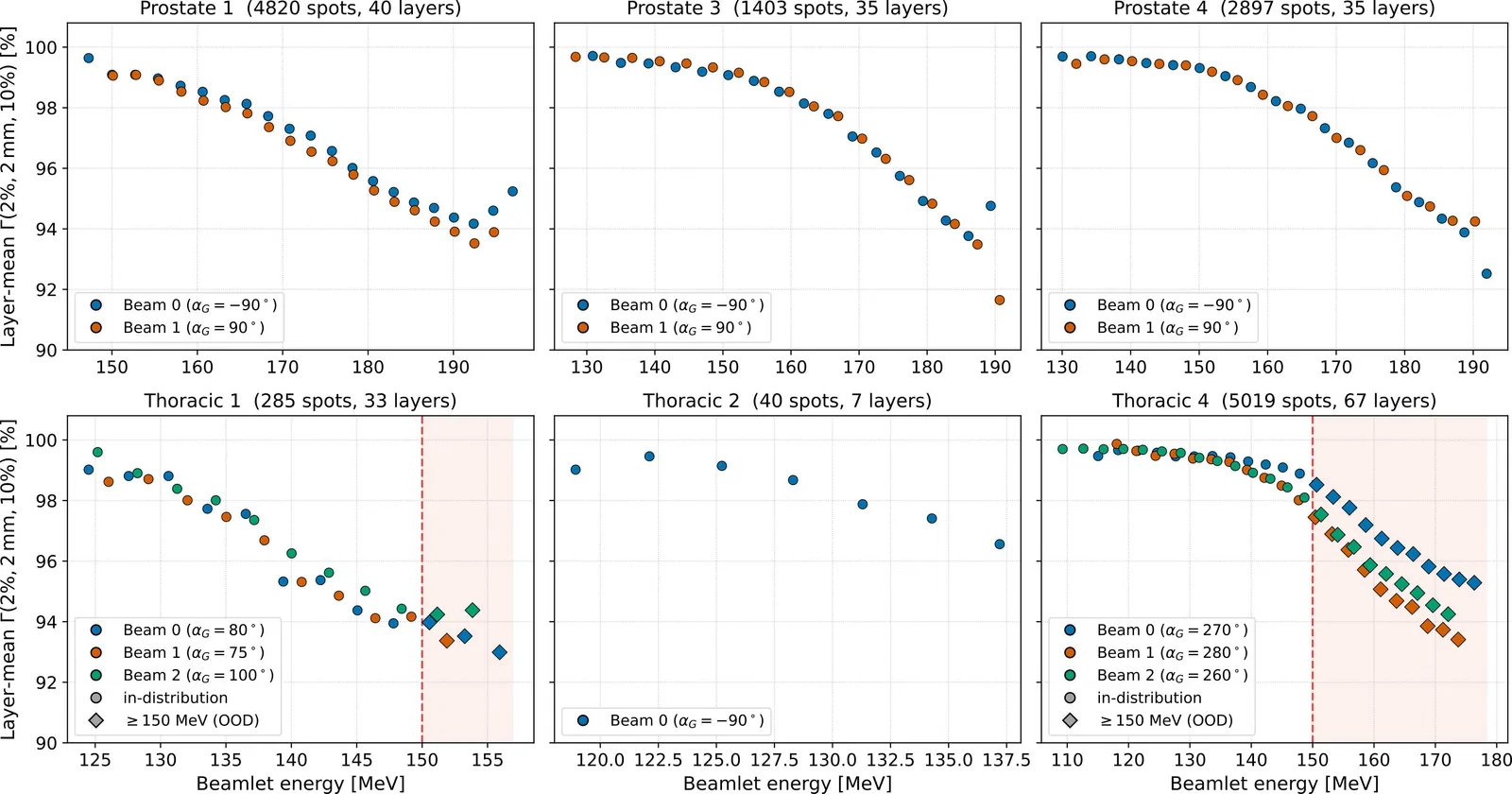
Layer-mean Γ(2%, 2 mm, 10%) pass rate versus beamlet energy, for 3 prostate plans (top row) and 3 thoracic plans (bottom row) from Table 2a. Each point is the mean gamma pass rate of one plan energy layer; panel titles state the number of beamlets and energy layers. Point color identifies the irradiation field (beam) within each plan (2 fields for the prostate plans and Thoracic 4, 3 for Thoracic 1, one for Thoracic 2), held consistent across panels, with each field’s gantry angle *α*_*G*_ stated in the legend; marker shape identifies whether that layer is in-distribution (circle) or at/above the 150 MeV beamlet-level training limit (diamond), with a shaded band marking the out-of-distribution (OOD) zone for the thoracic plans. No plan crosses the corresponding 270 MeV prostate limit.

## Data Availability

The main repository with the model architecture and auxiliary methods can be found at https://github.com/Medical-Physics-Technology/adota. The data that support the findings of this study are available from the authors upon reasonable request.

## Appendix A Additional Results

### Supplementary metrics

Complementary to the gamma pass rate (Γ) presented in Table 1, Table A1 reports the relative dose error (*ρ*) and root mean squared error (RMSE) benchmarked against original DoTA.^8^ Figure A1 illustrates the distributions of Γ(2%, 2 mm, 10%) and *ρ* on the reported individualized beamlet test set.

### Distribution of beamlet-level range errors

Table 1 reports the range metrics as signed means with their standard deviations. Because positive and negative errors partially cancel in a signed mean, Table A2 characterizes the distribution of Δ*R*_100_, Δ*R*_80_, Δ*R*_20_, and ΔDFW over the same 3500 beamlets: the MAE, the 5th, 50th, and 95th percentiles of the signed error, the 95th percentile and maximum of the absolute error, and the fraction of beamlets whose absolute error exceeds 2, 3, and 5 mm. The smallest reported threshold is 2 mm, matching the model’s native isotropic grid spacing.

The absolute-error and threshold statistics separate the 2 cohorts more sharply than the signed means do. For the clinical range metric Δ*R*_80_, no abdominal/pelvic beamlet exceeds a 3 mm absolute range error, whereas 10.0% of thoracic beamlets do and 5.4% exceed 5 mm. The thoracic distribution is also markedly heavier-tailed: the maximum absolute Δ*R*_80_ reaches 56.14 mm, against 2.96 mm in the abdominal/pelvic cohort. Such extreme values arise for beamlets traversing lung and air–tissue interfaces, where the laterally integrated depth-dose profile can flatten near the distal fall-off and the extracted range becomes correspondingly sensitive; a representative poorly-performing thoracic case is shown in Figure A16.

### Ablation study

Table A3 reports the 5 configurations described in Section “Training details”: the full ADoTA model, an MSE-only variant isolating the IDD loss term, a variant without the encoder–decoder convolutional residual connections, and 2 direction-encoding alternatives to the analytical projection **Φ** (Equation (2)), the constant-width Gaussian tube **Φ**^*E*^ (Equation (11)) and the binary centerline **Φ**^*F*^ (Equation (12)). The residual-connection ablation isolates that architectural modification relative to the prior DoTA model^8^ on our dataset; it does not reconstruct or retrain DoTA, which uses a different model and training corpus. All 5 configurations share identical training data, optimizer, schedule, and checkpoint-selection criterion (lowest validation MAPE) and are evaluated on the 14 029-beamlet validation set.

The 2 direction-encoding ablations differ only in how the beamlet centerline is rendered into the input channel. Both are defined in the beamlet-aligned frame (x′,y′,z′) of Equation (1), in which z′ is the depth along the central ray and $\left(x\prime_{c},y\prime_{c}\right)$ are the lateral coordinates; this frame is obtained from the gantry-rotated patient frame, so the opposite signs of *θ*_*x*_ and *θ*_*y*_ below follow the patient orientation convention of the DICOM standard. At depth z′ the central ray passes through the lateral position

(10) $$x\prime_{c}\left(z\prime \right)=x_{0}\prime +z\prime \tan\theta_{x},\;y\prime_{c}\left(z\prime \right)=y_{0}\prime -z\prime \tan\theta_{y},$$

where $\left(x_{0}\prime ,y_{0}\prime \right)$ is the beamlet entrance point. **Φ**^*E*^ is an isotropic Gaussian of fixed width *σ*_0_ = 3.4 mm, the dataset-median in-air spot size, centered on that position:

(11) $$\Phi^{E}\left(x\prime ,y\prime ,z\prime \right)=\exp\;\left(-\frac{\left(\right)x\prime -x\prime_{c}\left(z\prime \right){))}^{2}+\left(\right)y\prime -y\prime_{c}\left(z\prime \right){))}^{2}}{2\sigma_{0}^{2}}\right).$$

**Φ**^*F*^ marks, at each depth, the single voxel whose lateral center is nearest that position:

(12) $$\Phi^{F}\left(x\prime ,y\prime ,z\prime \right)=\left\{\begin{array}{cc} 1\; & \mathrm{if}\left(x\prime ,y\prime \right)=\left(\right)\mathrm{round}\left(x\prime_{c}\left(z\prime \right)\right),\;\mathrm{round}\left(y\prime_{c}\left(z\prime \right)\right))),\\ 0\; & \mathrm{otherwise,} \end{array}\right)$$

where round(⋅) denotes rounding to the nearest voxel center, so that exactly one lateral voxel is marked per depth slice.

All 5 configurations were trained and evaluated identically, using the same training data, optimizer, schedule, validation set, and checkpoint-selection criterion, and differing only in the component under ablation. For the 2 projection-encoding configurations, that component is the construction of the beamlet direction channel, which does not alter the evaluation protocol. Lower is better throughout.

### Robustness across varying penetration depths

To assess the model performance across different penetration depths, we calculated the mean gamma pass rate for each lateral depth slice of the dose volume. Figure A2a illustrates the stability of the gamma pass rate (Γ(1%, 3 mm, 0.1%)) as a function of beamlet penetration depth. Complementary to this, Figure A2b shows the percentage of test set samples exhibiting non-zero dose deposition at each corresponding depth. The gradual decline in the number of samples with increasing depth reflects the finite proton range in the geometry, as fewer beamlets penetrate to the deeper geometric layers. A related energy-domain analysis for the partially out-of-distribution thoracic plans is given in Appendix A.12.

### Extended angular robustness analysis

Figure A3, Figure A4, Figure A5, Figure A6 report the 36 individual panels underlying the aggregated cohort panels of Figure 4a-d: for each of the 6 patients of the population sweep (Section “Model evaluation”) and each of its 3 gantry angles, the Γ(2%, 2 mm, 10%) pass rate over the 18 × 18 grid of scanning angles. All 36 panels share a single common color scale. Cells are missing where a beamlet failed the FD≥0.99 retention criterion (Equation (3)), which occurs only in the thoracic geometries. Consistent with the aggregate view, within a given patient and energy the 3 independently drawn gantry angles agree to within approximately one percentage point, while the visible differences follow patient anatomy.

The angular sweep reported in Section “Results” was additionally extended to 2 further patient geometries, one abdominal/pelvic and one thoracic, distinct from the 6 patients of the population sweep, and to 3 additional beam energies (*ϵ* = 80, 120, and 140 MeV). The acquisition protocol was unchanged: the isocenter was placed at the image center, the CT grid was rotated by a randomly sampled gantry angle $\alpha_{G}\sim U\left(0^{\circ },360^{\circ }\right)$, and beamlets were scanned over an 18 × 18 grid of lateral angles *θ*_*x*_, *θ*_*y*_ ∈ [−2^∘^, 2^∘^], giving 324 scanned incidence angles per panel. A beamlet was retained only if its fractional dose FD (Equation (3)) reached the same 0.99 threshold used during training, that is, only if at least 99% of the dose it deposited on the full CT grid fell inside the cropped sub-volume presented to the model. At 140 MeV in the thoracic geometry, some beamlets overshoot the crop, fail this criterion, and were excluded; this is why that panel is not fully populated. All other panels retain the complete grid. Taken together with the population sweep, the angular evaluation therefore covers 8 patient geometries, 4 per anatomical site, at 5 distinct energies spanning 80 to 140 MeV, the lower end of which approaches the 70 MeV floor of the training range.

Figure A7 reports the extended sweep under the Γ(2%, 2 mm, 10%) criterion, and Figure A8 reports the same beamlets under the stricter Γ(1%, 3 mm, 0.1%) criterion. Within each figure all 6 panels share a single common color scale, so colors are directly comparable across anatomical sites and energies. Under Γ(2%, 2 mm, 10%), pass rates remain high across the full angular range in both cohorts, and the variation that is present tracks anatomical site and beam energy more strongly than beamlet incidence angle. It is clearest in the thoracic panel at 80 MeV (Figure A8d), which separates along the anti-diagonal into a low-scoring upper left half and a high-scoring lower right half. Its orientation differs between energies of the same geometry, so it does not follow the incidence angle. Appendix A.6 samples that anti-diagonal and attributes the variation to the traversed anatomy. As with the initial sweep, this remains an extended sensitivity analysis over a small number of geometries rather than a population-level generalization study.

The angular sweeps summarize each beamlet by a single pass rate. To show what the lower tail of those distributions corresponds to dosimetrically, Figures A9–A12 present the 2 worst-performing beamlets of each anatomical site, selected by Γ(2%, 2 mm, 10%) across all angles and energies of the extended sweep. Both thoracic cases occur at 80 MeV and both abdominal/pelvic cases at 140 MeV; the layout is identical to that of Figure A15.

### Influence of the traversed anatomy along the angular grid diagonal

The panels of Figure A8 show coherent structure within single geometries, which raises the question of whether it reflects sensitivity to the incidence angle itself or to the anatomy each incidence angle traverses. To separate the two, we sampled the anti-diagonal of the thoracic 80 MeV panel (Figure A8d), which shows the clearest contrast, at 18 positions running from (*θ*_*x*_, *θ*_*y*_) =(−2^∘^, +2^∘^) to (+2^∘^, −2^∘^) (Figure A13). Each position is one beamlet of the existing sweep, so no additional simulations were required.

Figure A14 tracks the traversed anatomy, the depth dose, and the prediction error along that line. The transect separates at position 10/11. Over positions 00–10 the beamlet crosses a long, lung-dominated path, with lung occupying up to 75% of the in-patient path length; the Bragg peak lies deep and broadens into a 21 to 31 mm dome at positions 05–07, ∣Δ*R*_80_∣ reaches 4.1 mm, and the pass rate stays between 80% and 89%. Over positions 11 to 17 the path is shorter and almost entirely soft tissue, with lung below 3% of the path; the distal fall-off is sharp, ∣Δ*R*_80_∣ stays below 0.5 mm, and the pass rate rises to 93% to 94%. Across the 18 positions the pass rate correlates with the lung fraction along the path (Spearman *ρ* = −0.87, *p* = 2.5 × 10^−6^), whereas the incidence angle itself varies monotonically through the transition without any corresponding change. The variation within the panel is therefore driven by the anatomy the beamlet traverses rather than by the incidence angle, consistent with the interpretation given in Section “Discussion.”

### Visual assessment of the ADoTA performance

Supplementary to Figure 3b and a, the worst- and best-performing samples from the abdominal/pelvic test set and the lung test set are presented in Figures A15–A18.

Supplementary to Table 2a, Figure A19 shows reconstructed full-plan dose distributions for plans **Prostate 2** and **Thoracic 4**, while Figures A20 and A21 show the DVHs for all 8 plans.

### Runtime evaluation

Table A4 consolidates 2 complementary views: (i) a cross-model comparison reporting the effective runtime per beamlet (including, where available, mean, standard deviation, and fastest/slowest values), and (ii) the ADoTA batch-scaling study reporting both batch forward-pass latency and the corresponding per-beamlet cost as a function of batch size. This batch analysis reflects the structure of proton therapy treatment planning, where plans are composed of many independent beamlets and the associated 3D dose evaluations can be efficiently parallelized via batched inference.

### Beamlet direction-encoding cost: GPU-optimized comparison

Table A5 reports the batch-size sweep underlying the ratios quoted in Sections “Methods and Materials” and “Discussion.” Before the 2 routes were compared, the CPU and GPU interpolators were verified to perform the same operation. Once the differing coordinate conventions are reconciled, voxel-index space against the normalized [−1,1] coordinates and the reversed axis order of grid_sample, SciPy affine_transform with order=1 and PyTorch grid_sample with bilinear interpolation and align_corners=True agree to within 10^−14^ at every angle tested. They differ only in out-of-bounds policy, which does not arise here because the sampling never leaves the crop at the angles used. No CPU timing is divided by a GPU timing: each reported ratio compares 2 routes measured on the same platform. All 5640 beamlets of the benchmark plan were used at every batch size. Each reported value is the mean over all units at that batch size, where a unit is one beamlet for the single-threaded CPU baseline and one batch for the GPU-batched cells, and each unit is itself the median of 5 repeated timings taken after one discarded warm-up call.

The analytical projection scales favorably with batch size, down to 0.076 ms per beamlet at batch 112 on compute, whereas the resampling plateaus around 0.25 to 0.35 ms once batched, consistent with the fixed per-launch overhead of grid_sample. The advantage, therefore, widens with batch size: at batch 1 the 2 routes are comparable on compute, and by batch 56 the analytical projection is 4.0× cheaper on compute and 6.9× cheaper including transfer, rising to 4.5× on compute at batch 112. Including host-device transfer, both routes reach their lowest per-beamlet cost at batch 32–56 and both rise at batch 112. This rise reflects the host destination buffer being remapped on every call once it exceeds the allocator’s 32 MiB threshold, not the transfer volume; reusing that buffer removes it, and it affects both routes equally. Batched on GPU, both routes are roughly 2 orders of magnitude faster than single-threaded on CPU (119× for the analytical projection and 192× for the resampling, against each route’s fastest compute-only cell), so neither side of the comparison is left unoptimized.

Table A6 reports the measured comparison on the full benchmark plan (5640 spots, 2 fields), each route run through the same streaming implementation used for full-plan reconstruction (Section “Treatment plans”), in which the CT is loaded once and beamlets are cropped, predicted, and accumulated in batches without writing intermediate results to disk, batched identically. The advantage has a single source: the DoTA-like route resamples every beamlet twice, once into the beam’s-eye-view frame and once back to the field-aligned grid, which costs 25.46 seconds over the plan, whereas the analytical projection replaces both passes for 2.64 seconds. Every other stage performs the same work in both routes. Those stages are not numerically identical, and the differences that remain all favor the DoTA-like route: ADoTA additionally normalizes the projection channel during Prep (1.61 seconds), and it carries a further 0.61 seconds in Crop, with every other common stage differing by at most 0.05 seconds. Summed, the common stages cost ADoTA 2.32 seconds more, reducing the 22.82 seconds saved on direction encoding to a 20.50 seconds net advantage.

The stages of Table A6 are defined as follows. Field rotation and de-rotation are one paired transform applied once per field and trilinear in both directions: the CT is resampled onto the 2 mm field-aligned grid before prediction, and the accumulated field dose is resampled back onto the reference CT grid afterwards. Crop extracts each beamlet sub-volume from the field-aligned grid. Prep assembles the network input for each spot: host-device transfer of the CT and projection crops, min-max normalization of the CT, the energy, and, for ADoTA only, the projection channel, followed by concatenation into 2 channels; it performs no cropping, resampling, or type conversion. Forward is the network evaluation. Post-processing transfers each prediction back to the host, inverts its normalization, and reorders its axes. Dose deposit accumulates each predicted beamlet, scaled by its spot weight, into the field-aligned scoring grid.

The DoTA-like route’s own direction-encoding channel is not charged in this comparison, since a single-channel DoTA model has none; this is a simplifying assumption stated explicitly, not a measured cost.

### Detailed per-plan dose-volume metrics

Table 2a reports the target near-minimum dose error Δ*D*_98%_ and the mean absolute organ-at-risk near-maximum dose error ∣Δ*D*_2%_∣ as a compact plan-level summary. Table A7 extends this to the full breakdown: absolute MC and ADoTA values in Gy(RBE) and the signed difference (ADoTA−MC, as a percentage of the prescribed dose) for the target near-minimum dose *D*_98%_, coverage dose *D*_95%_, and near-maximum dose *D*_2%_, and the mean dose *D*_mean_ of each organ at risk, reported by anatomical name. A dash indicates the structure is not present in that plan.

### Effect of inference precision (FP16 vs. FP32) on plan-level dose accuracy

The full-plan reconstruction pipeline evaluates network inference under torch.autocast mixed precision (Section “Treatment plans”), which reduces 32 bit floating point (FP32) operations to 16 bit floating point (FP16) where supported. To test whether this precision reduction contributes to the plan-level target-dose bias discussed in Section “Discussion,” we recomputed the full-plan dose for one representative prostate plan (Prostate 1) and one representative thoracic plan (Thoracic 1) with network inference forced to FP32, keeping the grid factor, that is the integer ratio between the dose-scoring grid spacing and the model’s native 2 mm grid, together with the batching and model checkpoint, identical to the FP16 run, and compared the 2 using the gamma criteria and error metrics of Section “Methods and Materials.” Table A8 reports the results.

Across both plans, every gamma criterion changes by at most 0.025 percentage points between FP32 and FP16, and RMSE and the relative dose error *ρ* each change by less than 0.01 in their respective units. These differences are far smaller than the plan-level biases reported in Table 2a, which reach several percent of prescription. We conclude that FP16 inference precision has a negligible dosimetric effect and is not a contributor to the observed negative target-dose bias. The runtime benefit is concentrated in the network forward pass itself, reduced by 33.8% for Prostate 1% and 15.2% for Thoracic 1, since the remaining pipeline stages (cropping, projection construction, dose deposition, and I/O) are not evaluated in mixed precision; the corresponding reduction in total streaming runtime is smaller, at 15.0% and 5.2%, respectively.

### Beamlet-level gamma pass rate versus beamlet energy

Figure A22 reports the layer-mean Γ(2%, 2 mm, 10%) pass rate as a function of beamlet energy for 6 of the 8 plans in Table 2a with per-spot beamlet metrics available (Prostate 2 and Thoracic 3 are not included; their per-spot data was not available on the compute cluster). Beams are grouped by irradiation field and energy layer and the mean of the per-spot gamma pass rate within that layer is plotted against that layer’s nominal energy, following the same per-spot gamma computation used in Table 1. For the thoracic plans, layers at or above the 150 MeV beamlet-level thoracic training limit (Section “Methods and Materials”) are marked and shaded; the prostate plans are trained up to 270 MeV and never approach that limit within the evaluated energy range, so no such marking applies to them.

The pass rate declines gradually with energy in all 6 panels, including the fully in-distribution prostate plans and Thoracic 2 (maximum 137 MeV). The decline is a trend rather than a strictly monotonic fall: layer-to-layer scatter is present throughout, and in 4 of the thirteen fields the lowest layer mean occurs before the highest-energy layer, which then recovers slightly. In Thoracic 1 and Thoracic 4, the decline continues across the 150 MeV boundary with no abrupt change at the boundary itself (mean layer Γ(2%, 2 mm, 10%): Thoracic 1, 96.1% → 93.8%; Thoracic 4, 99.1% → 96.4%). This matches the depth-dependent decline already reported for the full beamlet test set in Figure A2 a (Section A.4), since initial energy and Bragg-peak depth are directly related.

The pattern is therefore consistent with previously documented, gradual model behavior rather than a distinct out-of-distribution failure mode. It does not, however, establish in-distribution accuracy for beamlets above 150 MeV: the 8-plan evaluation remains a proof of concept for plan reconstruction, not a clinical validation (Section “Treatment plans”).
